# Amino Acid Deprivation-Induced Autophagy Requires Upregulation of DIRAS3 through Reduction of E2F1 and E2F4 Transcriptional Repression

**DOI:** 10.3390/cancers11050603

**Published:** 2019-04-30

**Authors:** Margie N. Sutton, Gilbert Y. Huang, Jinhua Zhou, Weiqun Mao, Robert Langley, Zhen Lu, Robert C. Bast

**Affiliations:** 1 Department of Experimental Therapeutics, The University of Texas M.D. Anderson Cancer Center, Houston, TX 77030, USA; mnsutton@mdanderson.org (M.N.S.); gilbert.y.huang@gmail.com (G.Y.H.); jsjhzh@126.com (J.Z.); Wmao@mdanderson.org (W.M.); 2 Department of Obstetrics and Gynecology, The First Affiliated Hospital of Soochow University, Suzhou 215006, China; 3 Office of Translational Research, The University of Texas M.D. Anderson Cancer Center, Houston, TX 77030, USA; rlangley@mdanderson.org

**Keywords:** DIRAS3, ARHI, autophagy, ovarian cancer, amino acid deprivation, tumor dormancy, mTOR, nutrient sensing

## Abstract

Failure to cure ovarian cancer relates to the persistence of dormant, drug-resistant cancer cells following surgery and chemotherapy. “Second look” surgery can detect small, poorly vascularized nodules of persistent ovarian cancer in ~50% of patients, where >80% are undergoing autophagy and express DIRAS3. Autophagy is one mechanism by which dormant cancer cells survive in nutrient poor environments. DIRAS3 is a tumor suppressor gene downregulated in >60% of primary ovarian cancers by genetic, epigenetic, transcriptional and post-transcriptional mechanisms, that upon re-expression can induce autophagy and dormancy in a xenograft model of ovarian cancer. We examined the expression of DIRAS3 and autophagy in ovarian cancer cells following nutrient deprivation and the mechanism by which they are upregulated. We have found that DIRAS3 mediates autophagy induced by amino acid starvation, where nutrient sensing by mTOR plays a central role. Withdrawal of amino acids downregulates mTOR, decreases binding of E2F1/4 to the DIRAS3 promoter, upregulates DIRAS3 and induces autophagy. By contrast, acute amino acid deprivation did not affect epigenetic regulation of DIRAS3 or expression of miRNAs that regulate DIRAS3. Under nutrient poor conditions DIRAS3 can be transcriptionally upregulated, inducing autophagy that could sustain dormant ovarian cancer cells.

## 1. Introduction

Despite progress in surgery and chemotherapy, ovarian cancer still proves lethal in 70% of cases, leading to the death of more than 14,000 women in the United States each year [1]. One of the most important factors contributing to poor outcomes is the persistence of dormant, drug-resistant cancer cells after primary cytoreductive surgery and combination chemotherapy. Despite normalization of CA125 and negative imaging following primary treatment, “second look” exploratory surgery of the abdominal cavity can detect small, quiescent, poorly vascularized nodules of persistent ovarian cancer on the peritoneal surface in ~50% of patients. After positive second look operations, persistent ovarian cancer may take months or years to become clinically evident, consistent with tumor dormancy. In more than 80% of positive second looks, persistent ovarian cancer cells express DIRAS3 and are undergoing autophagy [2].

DIRAS3 is a maternally imprinted gene that encodes a 26 kD GTPase with 50–60% homology to Ras, but with a 34 amino acid N-terminal extension that reverses Ras function. We found that DIRAS3 is downregulated in 60% of ovarian cancers, and was the most downregulated gene compared to normal ovarian epithelial cells [3]. Previous work from our group established that loss of expression from the paternal allele can occur genetically through loss of heterozygosity, epigenetically by hypermethylation of both alleles, transcriptionally by loss of E2F1 and E2F4 promoter binding or post-transcriptionally by loss of the HER RNA binding protein [4,5,6,7,8,9,10]. Re-expression of DIRAS3 blocks mutant K-Ras^G12V^ and H-Ras^G12V^ -induced transformation of 3T3 and MCF-7 cells, prevents cancer cell growth, inhibits motility, induces autophagy and establishes tumor dormancy in xenografts [11,12].

Autophagy is a highly conserved catabolic process in which organelles and long-lived intracellular proteins are engulfed by double membrane vesicles, termed autophagosomes. Lysosomes then fuse with autophagosomes, forming autophagolysosomes [13]. After acidification of the autophagolysosomes, proteases and lipases are activated that hydrolyze proteins and lipids, releasing amino acids and fatty acids that can be catabolized to produce energy. Depending upon the cellular context, induction of autophagy can either sustain or eliminate metabolically active cancer cells. In the short term, energy released by autophagy can facilitate survival of cancer cells in nutrient deprived microenvironments with inadequate blood supply. Persistent autophagy can, however, eliminate cancer cells. Enhanced mammary tumorigenesis has been observed in heterozygous *BECN1*^+/−^ mice with reduced expression of an essential component required for autophagy [14]. 

DIRAS3 is a critical regulator of autophagy, (1) inhibiting AKT signaling, downregulating mTOR and decreasing phosphorylation of ULK1 to induce autophagy, (2) displacing Bcl-2 from BECN1 to form the autophagy initiation complex, (3) inhibiting Ras/MAPK and PI3K, retaining FOXO3a in the nucleus to induce critical proteins that participate in autophagy (LC3, Atg4), and (4) facilitating fusion of autophagosomes and lysosomes through Rab7 [2,11,15].

Re-expression of DIRAS3 induces tumor dormancy in human ovarian cancer xenografts in immune deficient mice [11]. Our group has developed the first inducible xenograft model for tumor dormancy in human ovarian cancer using tet-inducible expression of DIRAS3, demonstrating that autophagy is required to sustain small avascular deposits of human ovarian cancer cells in a nutrient poor environment [11]. Treatment of dormant DIRAS3 expressing human ovarian cancer cells with chloroquine, a functional inhibitor of autophagy, delays the outgrowth of dormant ovarian cancer cells after downregulating DIRAS3 expression in this xenograft model [11]. This model appears to be clinically relevant in that it mimics observation at second look operations of persistent DIRAS3 positive cancer cells that are undergoing autophagy [2]. Whether or not this is dependent upon selection of a subset of pre-existing DIRAS3 expressing/autophagy positive cancer cells in the primary tumor or an adaptive phenotype that is conferred by a nutrient deprived, poorly vascularized tumor microenvironment remains to be elucidated. 

In this study we have sought to determine whether nutrient starvation or amino acid deprivation can enhance DIRAS3 expression and induce autophagy in human ovarian cancer cells, providing one mechanism by which DIRAS3 can be upregulated in positive second look tumor specimens. Inhibition of mTOR following amino acid deprivation results in dissociation of E2F1 and E2F4 from the DIRAS3 promoter, with subsequent proteosomal degradation of these transcription factors. Loss of E2F1 and E2F4 permits transcriptional upregulation of DIRAS3, a critical member of the autophagy pathway. Similarly, inhibition of mTOR with the pharmacological inhibitor, rapamycin, results in increased E2F1 and E2F4 degradation, transcriptional upregulation of DIRAS3, and DIRAS3-mediated autophagy. In both cases, the transcriptional upregulation of DIRAS3 results in a positive feedback loop in which increased expression of DIRAS3 further decreases PI3K signaling and downstream phosphorylation of mTOR, resulting in further induction of autophagy. These data provide insight into the mechanism by which dormant tumors found at “second look” may adapt to the tumor microenvironment by upregulating DIRAS3 and autophagy.

## 2. Results

**Figure 1:**
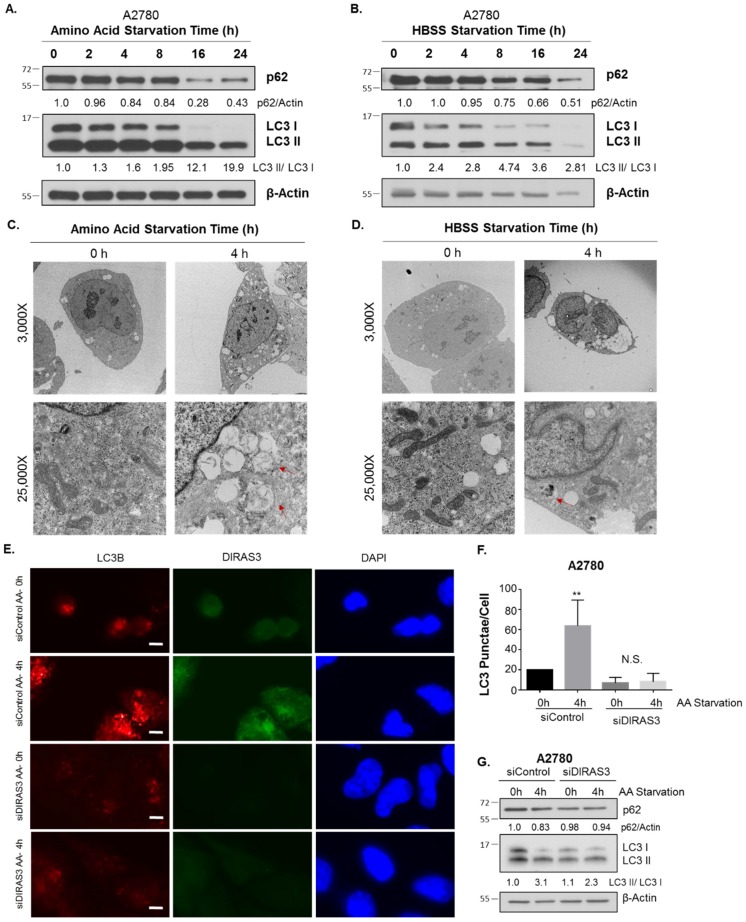
Starvation induces DIRAS3 mediated autophagy in A2780 ovarian cancer cells. A2780 ovarian cancer cells were treated from 0–24 h with amino acid free RPMI media (**A**) or HBSS containing 3% glucose (**B**) and western blot analysis performed. Densitometry was performed on the blots using ImageJ and calculations were normalized to the 0 h timepoint. Transmission electron micrographs were obtained following amino acid starvation (**C**) or HBSS Starvation (**D**) for 0 h or 4 h. Images were captured at 3000× and 25,000× magnification. Red arrows indicate typical double-membrane autophagosomes. (**E**) Immunofluorescence staining was performed following siRNA depletion of DIRAS3, with or without amino acid starvation for 4 h. LC3B puncta were visualized and quantified for each condition, as well as DIRAS3 expression and DAPI nuclear staining. Scale Bar represents 10 μm. Quantification of LC3B puncta was obtained for at least 100 cells over three independent experiments and is depicted in (**F**). (**G**) Western blot analysis was performed following siRNA depletion of DIRAS3, with or without amino acid deprivation for 4 h. Image J was used to quantify the band intensity and were normalized to the siRNA Control 0 h timepoint. Statistical significance is denoted as ** *p* < 0.01. Raw data films for western blot analysis shown in Figure S1.

**Figure A1:**
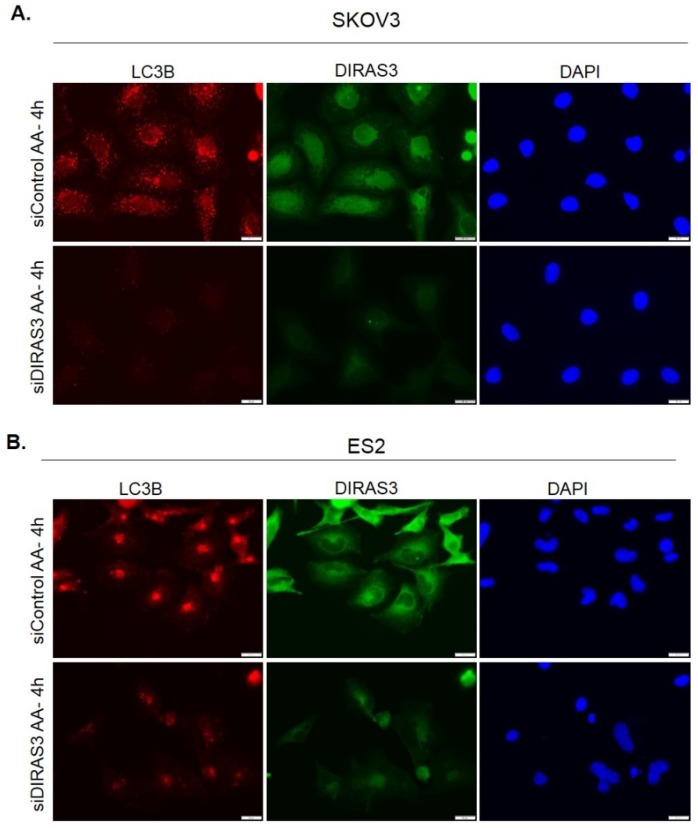
Knockdown of DIRAS3 inhibits starvation-induced autophagy. SKOV3 (**A**) or ES2 (**B**) ovarian cancer cells were treated with either control siRNA or siRNA directed at DIRAS3 for 72 h prior to undergoing amino acid deprivation for 4 h. Immunofluorescence staining was performed and LC3B puncta, DIRAS3 expression and DAPI nuclear staining were visualized. Representative images are shown for each condition. Scale bars indicate 20 μm.

Growth in media lacking amino acids with or without fetal bovine serum (FBS) induces DIRAS3-mediated autophagy in ovarian cancer cells. When A2780 ovarian cancer cells were grown in media lacking amino acids (amino acid starvation) or in serum-free Hanks buffered salt solution (HBSS) supplemented with 3% glucose for 0–24 h (serum starvation), western blot analysis demonstrated a decrease in p62 (SQSTRM) and conversion of LC3-I to LC3-II, two established markers of autophagy [16] (Figure 1A,B). Transmission electron microscopy (TEM) confirmed the induction of autophagy by both forms of nutrient deprivation after 4 h. Following amino acid depletion with or without FBS, classical autophagosomes with double membranes were observed in electron micrographs, associated with increased lipid membrane production and shrinking of cancer cells (Figure 1C,D). Treatment of A2780 ovarian cancer cells with DIRAS3 siRNA, but not control siRNA, significantly reduced (*p* < 0.05) induction of autophagy after amino acid starvation, demonstrated both by reduction of immunofluorescent LC3 puncta and western blot analysis (Figure 1E,G). Similar results were obtained with two additional ovarian cancer cell lines, SKOv3 and ES2 (Appendix A Figure A1). 

### 2.1. Amino Acid Deprivation Inhibits mTOR Signaling, Inducing Upregulation of DIRAS3 mRNA Expression and Autophagy

**Figure 2:**
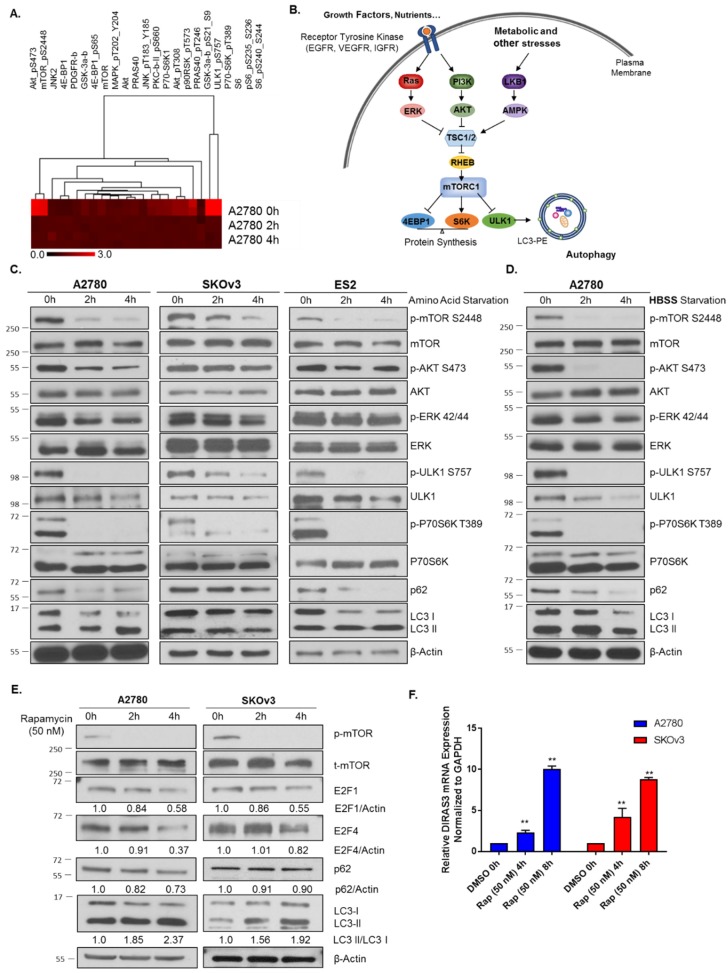
Nutrient deprivation leads to mTOR inhibition and activation of autophagy. (**A**) Reverse phase protein lysate arrays were performed following amino acid deprivation in A2780 cells for 2–4 h, in duplicate. A heat map was generated based on the fold change observed compared to unstarved cells. (**B**) Model depiction of key signaling pathways activated upon nutrient deprivation which can lead to autophagy induction. (**C**) Western blot analysis following amino acid starvation in A2780, SKOv3 and ES2 cells for 0–4 h. Blots were probed as indicated documenting a decrease in mTOR signaling, and induction of autophagy. (**D**) Western blot analysis following treatment of A270 ovarian cancer cells with HBSS supplemented with 3% Glucose for 0–4 h. Blots were probed as indicated and confirmed similar signaling and induction of autophagy as determined by RPPA analysis. (**E**) A2780 and SKOv3 ovarian cancer cells were treated with 50 nM Rapamycin for 0–4 h and western blot analysis performed. Blots were probed as indicated and decreases were observed in p-mTOR, E2F1 and E2F4 as well as the induction of autophagy. (**F**) qRT-PCR analysis following rapamycin (50 nM) treatment for 0–4 h. mRNA expression of DIRAS3 as compared to GAPDH was determined. Bars indicate the mean ± standard deviation. Experiments were performed in triplicate. **p < 0.01. Raw data films for western blot analysis shown in Figure S2.

The effects of nutrient deprivation on downstream signaling have been well documented and the convergence of mTORC signaling to regulate protein translation, proliferation, cell growth and autophagy puts it at the center of the stress response [17,18] (Figure 2B). Amino acid or serum starvation produced significant downregulation of both p-mTOR and p-P70S6K. Using reverse phase protein lysate arrays, we found many autophagy repressors were downregulated, including both the PI3K and Ras/MAPK signaling pathways, ULK1 p-S757, and p-8235/6 and p-8240/44 S6 kinase (Figure 2A,B). We confirmed these results, noting significant downregulation of p-AKT, p-ERK, p-mTOR, p-ULK1, p-P70S6K (Figure 2C) in A2780 ovarian cancer cells following both amino acid deprivation as well as complete serum starvation (HBSS supplemented with 3% glucose) at 2–4 h. As previously noted by Wang et al. [19], mTOR can regulate E2F1 expression, and inhibition of mTOR with the pharmacological agent, rapamycin led to reduced protein levels of E2F1 in non-small cell lung cancer cells [19]. To determine whether mTOR inhibition had a similar effect in ovarian cancer, we treated A2780 and SKOv3 cells with 50 nM rapamycin (as optimized for inhibition of mTOR without significant cell toxicity) for 4–8 h, and observed the effects on E2F1 and E2F4 protein expression and the corresponding *DIRAS3* mRNA expression. We found that inhibition of mTOR reduced E2F1 and E2F4 expression, increased *DIRAS3* mRNA, and induced autophagy as measured by LC3-I to LC3-II conversion (Figure 2E,F). 

### 2.2. Knockdown of Sestrin2 Suppresses Amino Acid Starvation-Induced Autophagy

**Figure 3:**
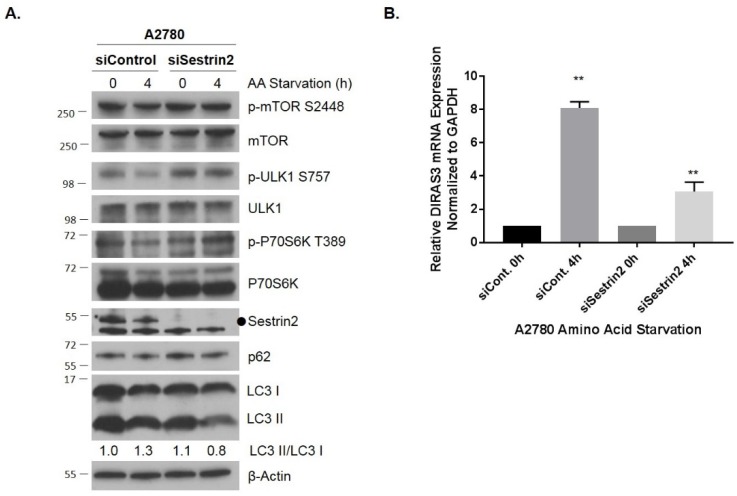
Knockdown of Sestrin2 inhibits amino acid starvation-induced mTOR inhibition, autophagy and upregulation of DIRAS3. A2780 cells were reversely transfected with control or Sestrin2 siRNA for 72 h prior to undergoing amino acid deprivation for 0–4 h. (**A**) Protein lysates were collected and western blot analysis was performed. Blots were probed as indicated. (**B**) qRT-PCR was performed and DIRAS3 mRNA was measured relative to GAPDH. The experiment was performed three independent times with technical duplicates. Bars indicated the mean ± the standard deviation; **p < 0.01. Raw data films for western blot analysis shown in Figure S3.

To test whether upregulation of DIRAS3-mediated autophagy is directly linked to a decrease in mTOR signaling following amino acid deprivation, we used siRNA to knockdown *Sestrin2*, a GATOR2-interacting protein that inhibits mTORC1 signaling in the absence of leucine [20,21]. Sestrin family proteins are highly conserved regulators of cell growth and survival in response to different stress conditions, that suppress oxidative stress and regulate adenosine monophosphate-dependent protein kinase (AMPK)-mammalian target of rapamycin (mTOR) signaling [20,21]. We found that following siRNA knockdown of the amino acid sensor, Sestrin2, amino acid deprivation at 4 h did not fully activate the mTOR signaling cascade, resulting in induction of autophagy and increased *DIRAS3* mRNA expression (Figure 3) compared to control siRNA, consistent with the possibility that induction of *DIRAS3* mRNA following amino acid deprivation is downstream of the effects on mTOR signaling. Interestingly, previous studies have demonstrated that re-expression of DIRAS3 can inhibit AKT/mTOR signaling, suggesting a positive feed-forward loop to further enhance the induction of autophagy following amino acid deprivation.

### 2.3. Acute Amino Acid Starvation Induces Transcriptional Upregulation of DIRAS3 with Decreased Binding of E2F1/E2F4 to the DIRAS3 Promoter and Decreased Levels of E2F1 and E2F4, But Does not Affect Methylation of the DIRAS3 Promoter or miRNA Regulation of DIRAS3 Expression

**Figure 4:**
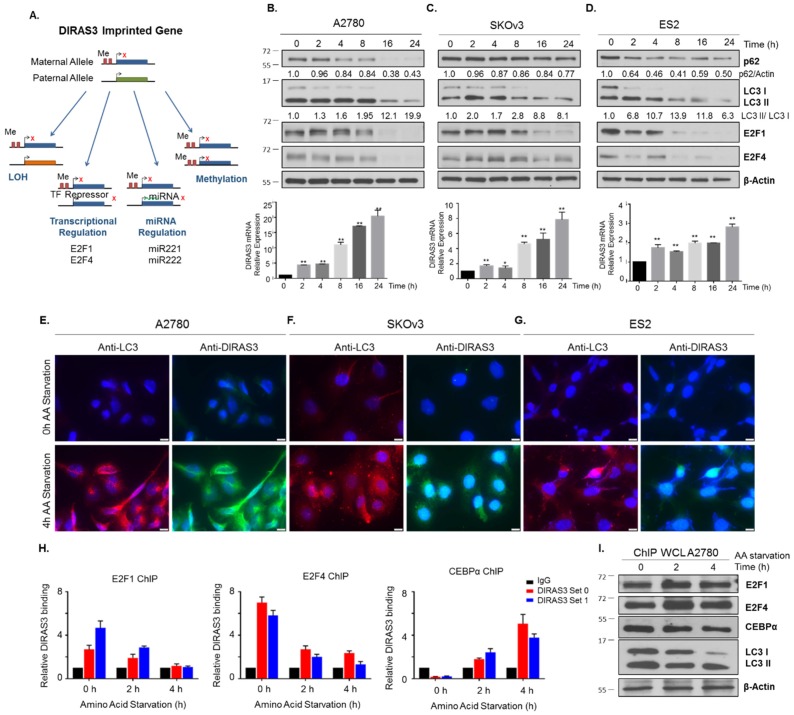
Amino acid deprivation induces DIRAS3 expression which correlates with an increase in autophagy. (**A**) DIRAS3 is a maternally imprinted tumor suppressor gene that is downregulated in ovarian cancer by several mechanisms including: LOH, transcriptional and miRNA regulation, and methylation of the non-imprinted allele. (**B**) A2780 (**C**) SKOV3 and (**D**) ES2 ovarian cancer cells were treated with amino acid free RPMI media from 0 h to 24 h. Protein lysate and RNA were collected for analysis. Western blots were performed to examine the expression of E2F1 and E2F4 transcription factors and autophagy markers p62 and LC3B. qRT-PCR was performed to determine the relative mRNA expression of DIRAS3 following amino acid deprivation. Immunofluorescence staining of (**E**) A2780 (**F**) SKOV3 and (**G**) ES2 cell lines confirmed upregulation of DIRAS3 expression and LC3B puncta following amino acid deprivation for 4 h. Scale bars indicate 20 nm. (**H**) Chromatin Immunoprecipitation was performed in A2780 ovarian cancer cells following amino acid deprivation for 0 h, 2 h and 4 h, using IgG, E2F1, E2F4 and CEBPalpha as the transcription factors of interest. Using two independent DIRAS3 primer sequences, and normalizing them to an IgG control, we examined the relative binding between E2F1, E2F4 or CEBPalpha and DIRAS3. Experiments were performed in technical triplicate over three independent experiments. Statistical significance was denoted as * *p* < 0.05 or ** *p* < 0.01. (**I**) Protein lysate from A2780 cells simultaneously treated was collected to determine the expression of E2F1, E2F4 and CEBPalpha following amino acid deprivation. Raw data films for western blot analysis shown in Figure S4.

In previous studies, we have found that DIRAS3 can be negatively regulated transcriptionally by binding of E2F1 and/or E2F4 to the *DIRAS3* promoter, epigenetically by DNA-hypermethylation of CpG islands in promoter regions of the imprinted and non-imprinted alleles of *DIRAS3*, and post-transcriptionally by the expression of miRNA-221 and miRNA-222 [4,5,9]. Upregulation of DIRAS3 might result from reversal of these processes during amino acid starvation. The E2F family of transcription factors have been well characterized for their role in regulating cell cycle progression, and recent reports have emerged documenting their context dependent role under different stresses, such as nutrient deprivation, and response to chemotherapeutic drugs [22]. Due to these differential responses, some E2F family members have been proposed as therapeutic targets or biomarkers for the prognosis of human lung cancers [23]. Using qRT-PCR to measure *DIRAS3* mRNA expression, we found that amino acid starvation in the presence or absence of serum increased *DIRAS3* mRNA in a time dependent manner (Figure 4A–G) with an initial elevation by 2 h. To determine whether upregulation of *DIRAS3* mRNA correlated with decreased binding of E2F1 and/or E2F4 to the *DIRAS3* promoter, we performed chromatin immunoprecipitation (ChIP) analysis after 2-4 h of amino acid deprivation. At these early intervals after amino acid starvation, DNA binding of both E2F1 and E2F4 to the *DIRAS3* promoter was significantly reduced (Figure 4H–J). At later intervals (4 h), E2F1 and/or E2F4 protein expression decreased. Downregulation of E2F1/4 correlated with the induction of autophagy and upregulation of DIRAS3 protein determined by immunofluorescence staining of LC3 puncta and DIRAS3 protein in A2780, SKOv3, and ES2 ovarian cancer cells (Figure 4E–G). 

**Figure A2:**
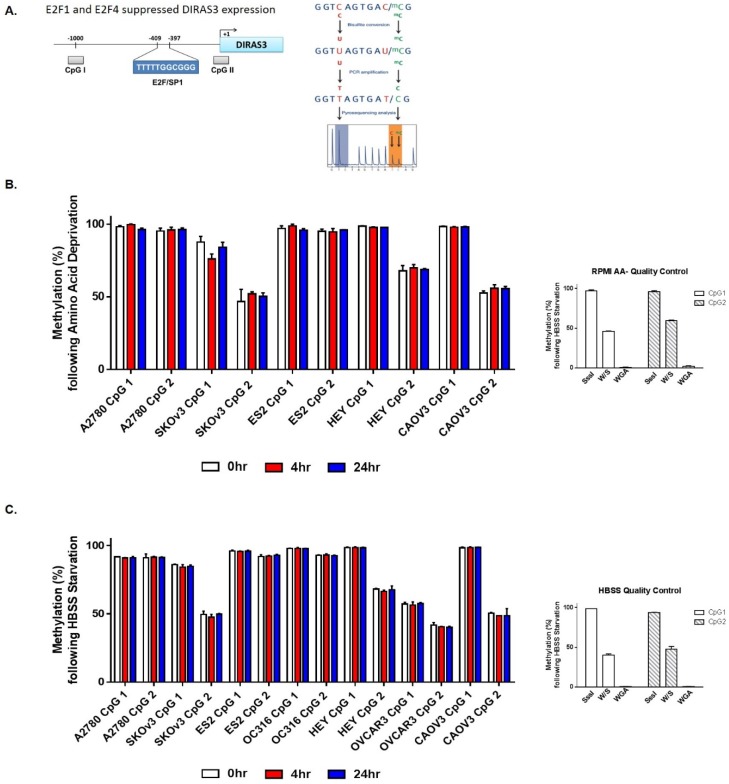
Methylation status of DIRAS3 does not change following amino acid deprivation. (**A**) Schematic depicting pyrosequencing process. Genomic DNA was collected following amino acid (**B**) or serum deprivation (**C**) for 0–24 h in several ovarian cancer cell line and pyrosequencing was performed. Graphs indicate the percent methylation at CpG island 1 or CpG island 2.

To determine whether acute amino acid starvation could induce demethylation of the *DIRAS3* promoter we performed pyrosequencing to determine the methylation status following amino acid deprivation in the presence and absence of serum for 4 h and 24 h. Based on sequencing of two distinct *DIRAS3* CpG Islands (Appendix A Figure A2), we did not observe a change in methylation status following nutrient deprivation that induced autophagy at early timepoints (Appendix A Figure A2). 

**Figure A3:**
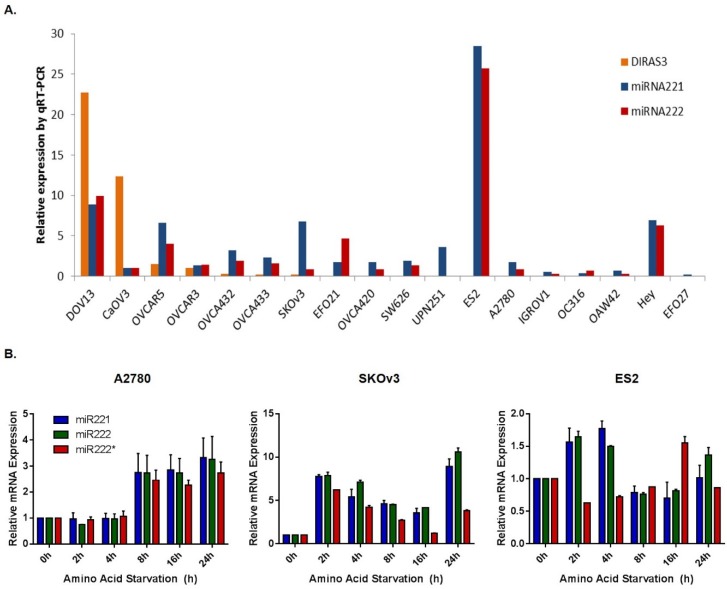
MicroRNA expression and profiling following amino acid deprivation in several ovarian cancer cell lines. (**A**) Basal miRNA expression of miR-221 and -222 was measured and compared to DIRAS3 mRNA expression across several ovarian cancer cell lines. (**B**) miRNA-221 and -222 expression was measured by qRT-PCR following amino acid deprivation for 0–24 h in A2780, SKOv3 and ES2 cells.

Similarly, we measured levels of several microRNAs previously reported to regulate DIRAS3 expression and their expression was not correlated over the 0–24 h during which DIRAS3 expression increased and autophagy was induced after amino acid deprivation (Appendix A Figure A3). This was especially true for miR222 in which two sets of primers (green and red) showed differential effects at early time points.

### 2.4. Knockdown of E2F1/E2F4 Induces DIRAS3-Mediated Autophagy

**Figure 5:**
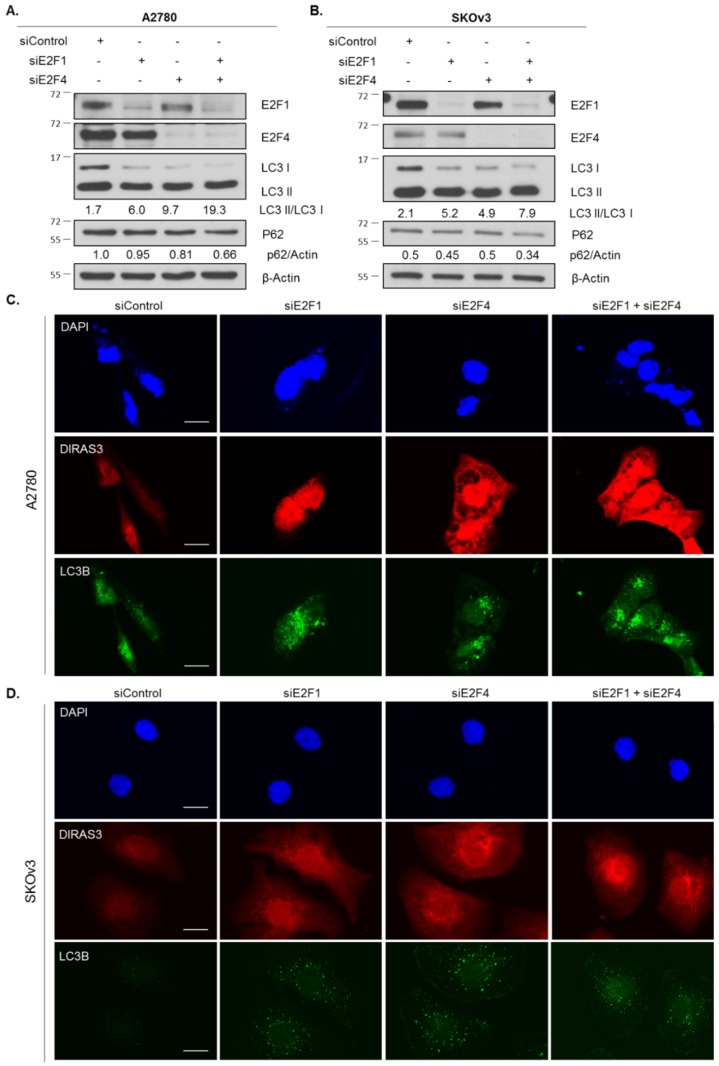
Knockdown of E2F1 or E2F4 phenocopies amino acid starvation-induced upregulation of DIRAS3-mediated autophagy. A2780 (**A**) and SKOv3 (**B**) ovarian cancer cells were treated with control or targeted siRNA for 72 h. Protein lysate was collected and western blot analysis was performed. Blots were probed as indicated. Immunofluorescence staining was performed on A2780 (**C**) and SKOv3 (**D**) cells following control or targeted siRNA treatment as previously described. DAPI was used to stain the nucleus. Scale bars indicate 15 µm (A2780 and 5 µm (SKOv3)). Raw data films for western blot analysis shown in Figure S5.

To test whether loss of E2F1 and E2F4 was linked directly to the induction of starvation-mediated autophagy, we performed siRNA knockdown of both transcription factors individually and in combination. Knockdown of *E2F1* in A2780 and SKOv3 ovarian cancer cells induced autophagy measured by LC3 conversion on western blot analysis and puncta formation by immunofluorescence staining. While knockdown of *E2F4* also increased autophagy, combined knockdown had no greater effect (Figure 5). As expected, the induction of autophagy corresponded with an increase in DIRAS3 protein expression determined by immunofluorescence staining (Figure 5C,D).

**Figure 6:**
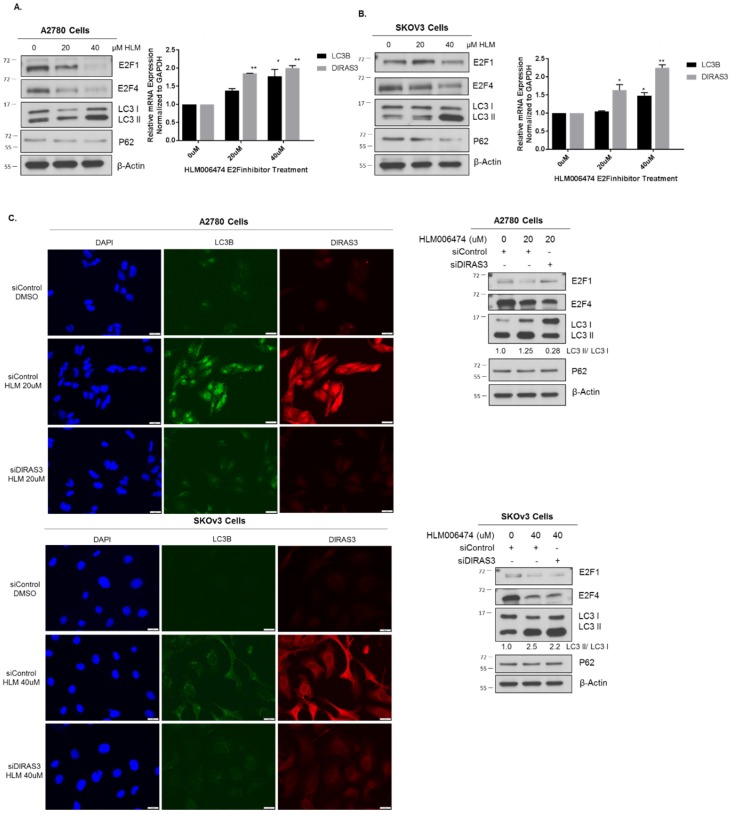
Pharmocologic inhibition of E2F1/4 induces DIRAS3-mediated autophagy. A2780 (**A**) or SKOv3 (**B**) ovarian cancer cells were seeded at 0.3 × 10^6^ cells/well in a 6-well plate and treated with 20–40 μM HLM006474 for 24 h. Protein lysate was collected for western blot analysis and probed as indicated. RNA was extracted and qRT-PCR was performed to measure DIRAS3 and MAPLC3B mRNA expression compared to GAPDH. The experiment was performed three independent times with technical duplicates. Bars indicated the mean ± the standard deviation; * *p* < 0.05 and ** *p* < 0.01. (C) A2780 and SKOv3 cells were treated with control or DIRAS3 siRNA for 48 hprior to treatment with 20–40 μM HLM006474 for 24 h. Immunofluorescence staining and western blot analysis was performed as indicated. Knockdown of DIRAS3 inhibited HLM006474-treatment induced autophagy. Raw data films for western blot analysis shown in Figure S6.

Treatment with an E2F inhibitor HLM06474 produced a significant increase in autophagy that could be diminished upon siRNA depletion of *DIRAS3* (Figure 6). Previous reports document that HLM06474 [24] not only inhibits E2F activity, but also decreases E2F4 protein expression. To test whether pharmacologic inhibition of E2F1 or E2F4 could induce DIRAS3-mediated autophagy, we treated A2780 and SKOv3 cells with 20–40 µM HLM06474 and observed a decrease in E2F1 and E2F4 protein expression, upregulation of *DIRAS3* mRNA and an increase in LC3 conversion on western blot (Figure 6A,B). To determine whether the induction of autophagy following E2F1/4 inhibition was DIRAS3 dependent, we treated A2780 and SKOv3 ovarian cancer cells with *DIRAS3* or control siRNA. A decrease in autophagic flux was observed in both cell lines judged by immunofluorescence and western blot analysis (Figure 6C). 

### 2.5. CEBPα Positively Regulates DIRAS3 Expression and Is also Required for Starvation-Induced DIRAS3-Mediated Autophagy

**Figure 7:**
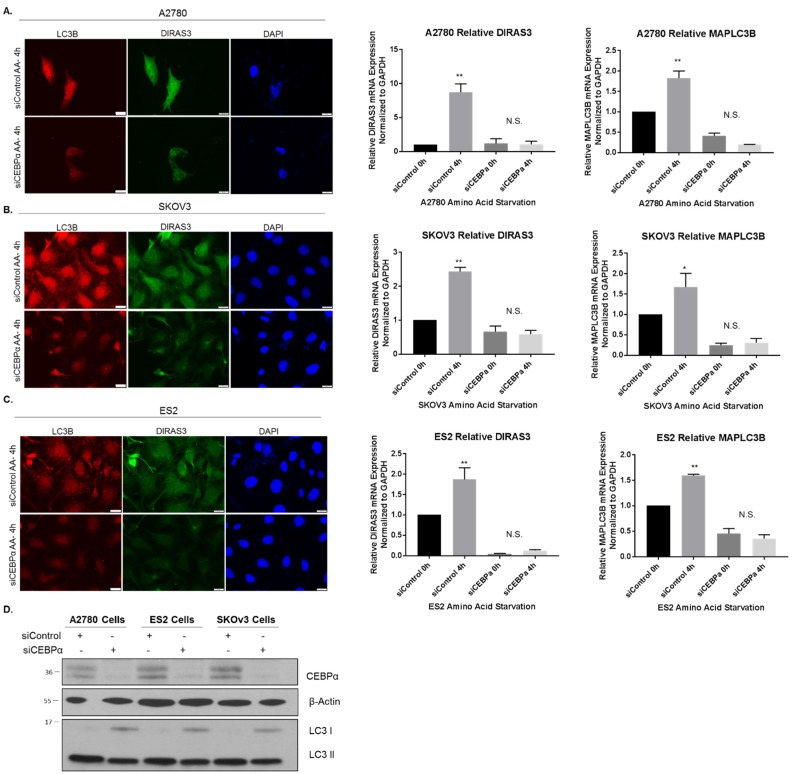
Knockdown of CEBPα reduces DIRAS3 upregulation following amino acid deprivation. A2780 (**A**) SKOv3 (**B**) and ES2 (**C**) ovarian cancer cells were treated with control siRNA or siRNA against CEBPα for 72 h prior to amino acid starvation for 4 h. Immunofluorescent staining was performed as indicated. qRT-PCR was performed and DIRAS3 and MAPLC3B mRNA expression was determined relative to GAPDH. The experiment was performed three independent times with technical duplicates. Bars indicated the mean ± the standard deviation; * *p* < 0.05 and ** *p* < 0.01. (**D**) Western blot analysis was performed to document the knockdown efficiency of CEBPα following siRNA treatment. Raw data films for western blot analysis shown in Figure S7.

CCAAT/enhancer-binding protein-α (CEBPα) is a transcription factor that can bind methylated DNA, and that can reduce E2F1 repression during adipocyte differentiation [25,26]. Others have also documented CEBPα protein in complexes bound at consensus E2F binding sites [27]. To determine whether CEBPα could act as a positive regulator of *DIRAS3* transcription during amino acid deprivation in A2780 ovarian cancer cells, we performed chromatin immunoprecipitation experiments following starvation for 2–4 h. Upon nutrient deprivation, increased levels of *DIRAS3* bound to CEBPα (Figure 4H). When CEBPα was knocked down with siRNA, we observed that nutrient deprivation was no longer able to upregulate *DIRAS3* mRNA expression across several ovarian cancer cell lines (Figure 7A–C) and this correlated with a decrease in autophagy induction as determined by immunofluorescence staining of LC3 puncta (Figure 7A–C). Interestingly, we observed that LC3 expression and mRNA levels were also decreased following siRNA knockdown of *CEBPα*, suggesting that *MAPLC3B* might be a transcriptional target of CEBPα as previously reported [28]. 

## 3. Discussion

Our findings document a requirement for DIRAS3 in establishing the autophagy induced by amino acid deprivation in the presence or absence of serum. Under nutrient poor conditions, DIRAS3 is upregulated transcriptionally by decreased binding of E2F1 and E2F4 and increased binding of CEBPα to the *DIRAS3* promoter. Both genetic and pharmacological inhibition of E2F1/4 family members enhance DIRAS3-mediated autophagy in ovarian cancer cells. While DIRAS3 levels can be regulated by promoter methylation and miRNAs [4,5,6,7,8,9,10], acute amino acid deprivation fails to affect either of these mechanisms by which DIRAS3 is traditionally silenced. 

Autophagy could be important for the persistence of dormant chemotherapy resistant ovarian cancer cells following primary surgery and chemotherapy. Previous work from our group reported that small deposits of tumor which remain in collagenous scars on the peritoneal cavity following primary chemotherapy and surgery, express DIRAS3 and undergo autophagy in more than 80% of cases, whereas primary cancers from the same patients express DIRAS3 and punctate LC3 in less than 20% of cases [2]. While it is possible that DIRAS3 expressing autophagic cells have been selected during primary treatment, the small fibrotic tumor nodules found on the peritoneal surface have few tumor associated vessels and it is likely that cancer cells are nutrient deprived. Our results suggest that amino acid or serum deprivation can induce DIRAS3 regulated autophagy, consistent with adaptation of persistent, drug resistant cancer cells to nutrient poor conditions. 

Although autophagy has long been documented as a normal cellular process that is essential for recycling of mitochondria, peroxisomes and long-lived proteins, its role in carcinogenesis, tumor progression and dormancy remains controversial [29]. This is largely due to the opposing, context-dependent roles that have been reported leading to the proposal of enhancing or inhibiting autophagy as a therapeutic strategy in cancer (Reviewed in references [30,31]). In nutrient- deprived ovarian cancer cells found in scars on the peritoneal surface, autophagy could provide energy to sustain cancer cells and facilitate tumor dormancy in the absence of tumor vessels [32,33]. Observations with a DIRAS3–induced model for ovarian cancer dormancy are consistent with this possibility. In this model, upregulation of DIRAS3 arrests cancer cell growth, prevents angiogenesis, and induces both autophagy and tumor dormancy. Treatment of dormant ovarian cancer cells with chloroquine, a functional inhibitor of autophagy, markedly delays outgrowth of tumors when dormancy is abrogated and growth permitted by downregulation of DIRAS3. Conversely, an excess of autophagy can lead to cancer cell death. DIRAS3-induced autophagic cancer cell death could be one mechanism that maintains the balance of cancer cell proliferation and cancer cell death, resulting in the ‘dormant’ phenotype. This hypothesis is supported by the work of Qiu et al. [34] where they reported that overexpression of DIRAS3 in gastric cancer cells inhibited metastasis in vivo, and correlated with increased autophagy [34]. 

In either case, autophagy could provide a therapeutic target for eliminating dormant, drug resistant ovarian cancer cells that remain after primary surgery and chemotherapy. Inhibition of autophagy has been achieved clinically by treatment with hydroxychloroquine with encouraging results in colorectal and pancreatic cancer [35,36], although the pharmacokinetics hydroxychloroquine is at the limit of concentrations required for effective inhibition of autophagy. Dimeric chloroquines and quinocrines have been shown to be 100- to 1000-fold more potent than hydroxychloroquine [37] and may provide additional benefit. Conversely, autophagic cancer cell death could be augmented by eliminating survival factors secreted by ovarian cancer cells or found in the tumor microenvironment including IGF, IL-8 and VEGF. Treatment of mice with dormant autophagic human ovarian cancer xenografts using antibodies against VEGF, IL-8 and the IGF receptor significantly prolonged survival and cured a majority of mice [38]. 

Another novel observation is that the CEBPα transcription factor positively regulates DIRAS3 expression and is also required for starvation-induced DIRAS3-mediated autophagy. Recently, emerging evidence has changed the classical dogma that transcription factor-DNA binding does not occur when high levels of methylation at CpG dinucleotides (mCpG) are present in the cis-regulatory region except for a few proteins which contain a mCpG-binding domain (MBD) [39,40]. Previous studies reported that a select few transcription factors without MBDs were found to bind methylated DNA with high affinity for specific sequences, but recent reports using systematic approaches have revealed that the number of transcription factors capable of binding and activating transcription of methylated DNA is much larger [39] and CEBPα is one of the factors on that list.

## 4. Materials and Methods

### 4.1. Antibodies and Reagents

Antibodies against LC3 (2775S), β-actin (4910L), pAkt^S473^ (9271S), AKT (9272), mTOR (2972), p-mTOR^S2448^ (2971), E2F1 (3742), p-P70S6K^T389^ (9205), P70S6K (2708), p-ULK1^S757^ (6888), ULK1 (4773) p-ERK^T202/Y204^ (4370), ERK (4695) were purchased from Cell Signaling Technology (Danvers, MA, USA). Antibodies against DIRAS3 (ARHI) were generated in our laboratory, and do not show cross reactivity with other members of the DIRAS family. P62/SQSTM1 antibody was purchased from MBL (Woburn, MA, USA). Anti-E2F4 (10923-1-AP) antibody was purchased from proteintech (Rosemont, IL, USA). Anti-CEBPα D-5 (sc-365318), anti-AKT (sc-5298) and anti-ERK (sc-514302) antibodies was purchased from Santa Cruz Biotechnology (Santa Cruz, CA, USA).

siRNAs were purchased from Dharmacon (Lafayette, CO, USA) and used as suggested by the manufacturer. E2F1 (1869) E2F4 (1874) DIRAS3 (9077) Sestrin2 (83667) CEBPα (1050) Non-targeting control (1810).

### 4.2. Cell Lines

Human ovarian cancer cells, A2780, SKOv3, ES2 and OVCAR8 were grown in RPMI medium, supplemented with 10% FBS and 1% L-glutamine. Amino acid free media was prepared from RPMI powder (US Biological R8999-04A) supplemented with 10% dialyzed (MWCO 10k) FBS (26400-044). Full starvation media was generated with HBSS supplemented with 3% glucose. Cells were routinely checked for mycoplasma contamination.

### 4.3. Measurement of mRNA Expression

Cells were seeded in a 6-well plate at 1.5 × 10^5^ cells/well, or following reverse transfection of siRNA at 1.0 × 10^5^ cells/well, and treated as indicated. RNA was extracted with Aurum Total RNA Mini Kit (BioRad #7326820, Hercules, CA, USA). 200–400 ng of RNA was used in a SYBR green based quantitative PCR (One-Step RT-qPCR from RNA, BioRad #1725150, Hercules, CA, USA) to measure RNA levels. Relative expression was calculated by the 2^−ΔΔCT^ method using glyceraldehyde-3-phosphatase dehydrogenase *(GAPDH)* as the reference gene. The experiments were repeated a minimum of three times, and samples were measured in technical duplicate. 

Primers included (5′ → 3′):
GAPDHForwardATGGAATCCATCACCATCTTReverseCGCCCCACTTGATTTTGGLC3Forward 356FGAAGCAGCTTCCTGTTCTGGReverse 588RTCATCCCGAACGTCTCCTGGDIRAS3Forward GTACCTGCCGACCATTGAAAAReverse GGGTTTCCTTCTTGGTCACTGE2F1Forward 734FACGTGACGTGTCAGGACCTReverse 879RGATCGGGCCTTGTTTGCTCTTE2F4Forward 202FATCGGGCTAATCGAGAAAAAGTCReverse 350RTGCTGGTCTAGTTCTTGCTCC

### 4.4. Brightfield and Fluorescence Microscopy

Microscopy was performed using an Olympus IX71 microscope equipped with a DP72 camera and an XM10 camera at the specified magnifications (Shinjuku, Tokyo, Japan).

### 4.5. Transmission Electron Microscopy

Cells were seeded at (1.0–2.5 × 10^5^ cells/well) in a 6-well plate and incubated for different intervals in amino acid-free medium. Samples were then fixed with Karnovsky’s fixative solution containing 3% glutaraldehyde plus 2% paraformaldehyde in 0.1 M cacodylate buffer at pH 7.3 and stored at 4 °C. After fixation, samples were submitted to the MD Anderson electron microscopy core facility for processing. Briefly, cells were washed in 0.1 M cacodylate buffer and treated with 0.1% Millipore-filtered buffered tannic acid, postfixed with 1% buffered osmium tetroxide for 30 min, and stained with 1% Millipore-filtered (0.2 μM) uranyl acetate. The samples were washed several times in water and then dehydrated in increasing concentrations of ethanol, infiltrated, and embedded in LX-112 medium. The samples were polymerized in a 60 °C oven for 2 days. Ultrathin sections were obtained using a Leica Ultracut microtome (Leica, Wetzlar, Germany), stained with uranyl acetate and lead citrate in a Leica EM Stainer, and examined in a JEM 1010 transmission electron microscope (JEOL USA Inc., Peabody, MA, USA) at an accelerating voltage of 80 kV. Digital images were obtained using AMT Imaging System (Advanced Microscopy Techniques Corp, Woburn, MA, USA).

### 4.6. Western Blotting

Cell lysates were prepared as indicated following incubation in lysis buffer (50 mM Hepes, pH 7.0, 150 mM NaCl, 1.5 mM MgCl_2_, 1 mM EGTA, 10 mM NaF, 10 mM sodium pyrophosphate, 10% glycerol, 1% Triton X-100) plus protease and phosphatase inhibitors (1 mM PMSF, 10 µg/mL leupeptin, 10 µg/mL aprotinin and 1 mM Na_3_VO_4_). Cells were lysed for 30 min on ice, and then centrifuged at 17,000× *g* for 30 min at 4 °C. The protein concentration was assessed using a bicinchoninic acid (BCA) protein assay (ThermoScientific, #23225, Waltham, MA, USA). Equal amounts of protein were separated by 8–16% SDS-PAGE, transferred to PVDF membranes and subjected to western blotting using an ECL chemiluminescence reagent (PerkinElmer, #NEL105001, Waltham, MA, USA).

### 4.7. siRNA Transfection

Cells were transfected with control or DIRAS family siRNAs (single and pooled oligos) using the Transfection #1 or #4 reagent (Dharmacon Research, #T-2001-01, #T-2004-01, Lafayette, CO, USA). Briefly, a mixture of siRNA (100 nM final concentration) and transfection reagents were incubated for 20 min at room temperature. This mixture was then added to cells and allowed to incubate for 48–72 h before cells were harvested for analysis.

### 4.8. Immunofluorescent Staining

Tumor cells (3 × 10^4^) were seeded in chamber slides and treated as specified. Cells were fixed with 4% paraformaldehyde and permeabilized with 0.5% Triton X-100. Cells were washed with PBS, and blocked with 5% BSA/PBS followed by incubation with the primary antibody. After washing, cells were incubated with secondary antibodies conjugated with Alexa Fluor 488 or 594 (Molecular Probes, #A11017, #A11020, #A11070, #A11072), mounted and examined using a fluorescence microscope (Olympus IX71 microscope with an XM10 camera).

### 4.9. Quantitative Pyrosequencing

Methylation Analysis of the DIRAS3 CpG Islands. Genomic DNA was extracted from ovarian cancer cell lines following treatment with HBSS plus 3% glucose or amino acid free RPMI media for 0 h, 4 h and 24 h, using the QIAamp DNA Mini extraction kit. (Qiagen #51304, Lot 157030756, Hilden, Germany) 1ug of genomic DNA was treated with sodium bisulfite using the EZ DNA Methylation-Gold Kit (Zymo Research, Irvine, CA, USA) according to the manufacturer’s protocol. The samples were eluted in 40 μL of M-Elution Buffer, and 1 μL was used for each PCR reaction. Both bisulfite conversion and subsequent pyrosequencing analysis were done at the DNA Methylation Analysis Core, The University of Texas M.D. Anderson Cancer Center [41]. 

PCR primers for pyrosequencing methylation analysis of the genomic area proximal to the transcription start site of DIRAS3 were designed using the Pyromark Assay Design SW 2.0 software (Qiagen, Hilden, Germany).

DIRAS3-1-FGTAAGGGAGAAAGAAGTTAGADIRAS3-1-Rbiotin-TACTATCCTAACAAAACCCTCDIRAS3-1-SF2ATTTGGAAAAGGGATTGG

Sequence to Analyze: YGGTGGGAGGYGTAGAGGGAAAAAGGAAYGATATAATYGGGTTTTTTAG

PCR size: 184bp

DIRAS3-2-FGTTGGGTTAGTTTTTTATAGTTGGTTDIRAS3-2-Rbiotin-AACCAAACAACCTAAAAAACAAATACDIRAS3-2-SF2TTGGGGTGTTTAGTTGGTTG

Sequence to Analyze: TYGYGGTAGTTTTTTYGAGTAGYGTATTTG

PCR size: 207bp

In brief, a sequencing primer was identified within 1 to 5 base pairs near the CpG sites of interest, with an annealing temperature of 40 ± 5 °C. After that, forward and reverse primers are identified uspstream and downstream to the sequencing primer. Optimal annealing temperatures for each of these primers were tested using gradient PCR. Controls for high methylation (SssI-treated DNA), low methylation (WGA-amplified DNA), partial methylation (S/W) and no-DNA template were included in each reaction. PCR reactions were performed in a total volume of 15 µl using ZymoTaq Polymerase kit reagent (Zymo Research, Irvine, CA, USA), and the entire volume was used for each pyrosequencing reaction as previously described (Estecio et al. 2007 [41]). Briefly, PCR product purification was conducted with streptavidin-sepharose high-performance beads (GE Healthcare Life Sciences, Piscataway, NJ), and co-denaturation of the biotinylated PCR products and sequencing primer (3.6 pmol/reaction) was conducted following the PSQ96 sample preparation guide. Sequencing was performed on a PSQ HS 96 system (Biotage AB, Uppsala, Sweden) with the PyroMark Gold Q96 CDT Reagents (Qiagen, Hilden, Germany) according to the manufacturer’s instructions. The degree of methylation was calculated using the PyroMark CpG SW 1.0 software (Biotage AB, Uppsala, Sweden).

### 4.10. Chromatin Immunoprecipitation (ChIP) Assay

A2780 ovarian cancer cells were seeded in 15 cm dishes at 80% confluency, and treated with amino acid free media for 0–4 h. An extra plate was used to determine cell number and viability following treatment, and an additional plate was prepared for chromatin immunoprecipitation. Using the protocol and reagents provided by Millipore (EZ-Magna ChIP A #17-408, Burlington, MA, USA), 1 × 10^7^ cells were used for the assay. Sonication was performed on ice for 10 seconds at maximum intensity and resting for 2 min on ice between cycles for a total of 20–25 cycles. Once shearing of DNA was confirmed by agarose gel, lysate was mixed with 20 µL fully suspended Protein A magnetic beads, and 8 µg of antibody (IgG, E2F1, E2F4, CEBPα) per tube and allowed to rotate overnight at 4 °C. Immunoprecipitated DNA was purified using the spin columns provided and qRT-PCR was performed as described below. Each qPCR plate was performed in triplicate and GAPDH was used as a positive control for qPCR reaction. Analysis was performed to determine the relative DIRAS3 binding compared to the IgG negative control for each condition. 

qPCR reagent assembly for 1 reaction:
ddH_2_O3.6 µLiTaqSYBR-Green master Mix (BioRad #172-5124)10 µLPrimer Mix (10 µM working concentration F+R)0.4 µLDNA (20 µL diluted with 38 µL ddH_2_O)6.0 µL

qPCR Parameters:
Initial Denaturation94 °C10 minDenature94 °C20 sAnneal and Extension60 °C1.0 min

Repeat 60 cycles

Melt temperature curve

Primers included (5′ → 3′):
DIRAS3-0ChIP Forward 524FTTTACCGGTCTTGCCACTAATGChIP Reverse 341RTCCAAAAGCAGTTTAATGCAGGDIRAS3-1ChIP Forward 76FTTAAGAACCTTTTGCCTAGCCChIP Reverse 227RCGGTCCAACTGATTTTAGACGAA

### 4.11. miRNA Quantitative Reverse Transcription-Polymerase Chain Reaction (qRT-PCR)

Total RNA was extracted using TRIzol reagent (Invitrogen, Carlsbad, CA, USA) according to the manufacturer’s suggested protocol. RNA was further purified with the miRNeasy Mini Kit from Qiagen (217004). RNA purity was assessed by measuring absorption at 260 nm (A260) and at 280 nm (A280) and the ratio of A260/A280 was determined. Those with a ratio of 1.9–2.1 were considered acceptable. qRT-PCR was performed to assess miR-221, miR-222, miR-222*, and miR-181 levels following amino acid deprivation or basal levels within the cell lines using the CFX Connect Real-Time PCR Detection system from BioRad (Hercules, CA, USA). Total RNA was reversely transcribed into complementary DNA (cDNA) with specific stem-loop RT primers and a cDNA synthesis kit (BioRad). Hsa-miR-221, 222, 222*, and 181a-c were purchased from ABI (Assay IDs 000524, 002276, 002097, 000480, 001098, 000482). Amplifications were carried out in triplicate on MicroAmp optical 96-well microliter plates (BioRad MLL9651). Thermal cycling conditions were as follows: 95 °C for 5 min, followed by 40 cycles of 95 °C for 10 s and 59 °C for 40 s. U6 RNA was used as an internal control in all qRT-PCR assays for miRNA. The ΔΔCT method was used to compare the relative expression levels at different timepoints following amino acid deprivation, and the final PCR results were expressed as the relative expression compared to individual control samples in each assay.

### 4.12. Statistics

All experiments were repeated independently at least two times and the data (bar graphs) expressed as mean ± s.d., unless noted otherwise. Statistical analysis was performed using Student’s *t*-test (two-sample assuming unequal variances). The criterion for statistical significance was taken as *p* < 0.05 (two-sided).

## 5. Conclusions

Nutrient deprivation, including loss of amino acids, can cause transcriptional upregulation of DIRAS3 and DIRAS3-mediated autophagy. Although DIRAS3 is an imprinted tumor suppressor gene that is downregulated in ovarian cancer, our previous studies found that matched patient samples where positive second look tumors were removed documented that both DIRAS3 and autophagy were upregulated in the small avascular deposits. We identified that upon amino acid starvation, E2F1 and E2F4 transcriptional repressors of DIRAS3 are downregulated. By contrast, amino acid deprivation did not affect epigenetic regulation of DIRAS3 or expression of miRNAs that are known regulators of DIRAS3 expression. mTOR plays a central role in nutrient sensing, which not only strongly activates autophagy, but also mediates transcriptional upregulation of DIRAS3. Our studies demonstrate that DIRAS3 mediates autophagy induced by nutrient deprivation, and could be a critical mechanism by which persistent, drug resistant cancer cells adapt to nutrient poor conditions.

## Supplementary Materials

The following are available online at https://www.mdpi.com/2072-6694/11/5/603/s1. Raw data films for western blot analysis shown in Figures S1–S7.

## References

[1] Siegel R.L., Miller K.D., Jemal A. (2018). Cancer statistics, 2018. CA Cancer J. Clin..

[2] Lu Z., Baquero M.T., Yang H., Yang M., Reger A.S., Kim C., Levine D.A., Clarke C.H., Liao W.S., Bast R.C. (2014). DIRAS3 regulates the autophagosome initiation complex in dormant ovarian cancer cells. Autophagy.

[3] Yu Y., Xu F., Peng H., Fang X., Zhao S., Li Y., Cuevas B., Kuo W.L., Gray J.W., Siciliano M. (1999). *NOEY2* (ARHI), an imprinted putative tumor suppressor gene in ovarian and breast carcinomas. Proc. Natl. Acad. Sci. USA.

[4] Feng W., Lu Z., Luo R.Z., Zhang X., Seto E., Liao W.S., Yu Y. (2007). Multiple histone deacetylases repress tumor suppressor gene ARHI in breast cancer. Int. J. Cancer.

[5] Feng W., Marquez R.T., Lu Z., Liu J., Lu K.H., Issa J.P., Fishman D.M., Yu Y., Bast R.C. (2008). Imprinted tumor suppressor genes ARHI and PEG3 are the most frequently down-regulated in human ovarian cancers by loss of heterozygosity and promoter methylation. Cancer.

[6] Fujii S. (2003). Reactivation of the silenced and imprinted alleles of ARHI is associated with increased histone H3 acetylation and decreased histone H3 lysine 9 methylation. Hum. Mol. Genet..

[7] Yu Y., Fujii S., Yuan J., Luo R.Z., Wang L., Bao J., Kadota M., Oshimura M., Dent S.R., Issa J.P. (2003). Epigenetic regulation of ARHI in breast and ovarian cancer cells. Ann. N. Y. Acad. Sci..

[8] Yuan J., Luo R.Z., Fujii S., Wang L., Hu W., Andreeff M., Pan Y., Kadota M., Oshimura M., Sahin A.A. (2003). Aberrant methylation and silencing of ARHI, an imprinted tumor suppressor gene in which the function is lost in breast cancers. Cancer Res..

[9] Lu Z., Luo R.Z., Peng H., Huang M., Nishmoto A., Hunt K.K., Helin K., Liao W.S., Yu Y. (2006). E2F-HDAC complexes negatively regulate the tumor suppressor gene ARHI in breast cancer. Oncogene.

[10] Lu Z., Luo R.Z., Peng H., Rosen D.G., Atkinson E.N., Warneke C., Huang M., Nishmoto A., Liu J., Liao W.S. (2006). Transcriptional and posttranscriptional down-regulation of the imprinted tumor suppressor gene ARHI (DRAS3) in ovarian cancer. Clin. Cancer Res..

[11] Lu Z., Luo R.Z., Lu Y., Zhang X., Yu Q., Khare S., Kondo S., Kondo Y., Yu Y., Mills G.B. (2008). The tumor suppressor gene ARHI regulates autophagy and tumor dormancy in human ovarian cancer cells. J. Clin. Investig..

[12] Sutton M.N. (2017). The Role of the DIRAS Family Members in Regulating Ras Function, Cancer Growth and Autophagy. https://digitalcommons.library.tmc.edu/utgsbs_dissertations/741/.

[13] Levine B., Klionsky D.J. (2004). Development by self-digestion: molecular mechanisms and biological functions of autophagy. Dev. Cell.

[14] Yue Z., Jin S., Yang C., Levine A.J., Heintz N. (2003). Beclin 1, an autophagy gene essential for early embryonic development, is a haploinsufficient tumor suppressor. Proc. Natl. Acad. Sci. USA.

[15] Lu Z., Yang H., Sutton M.N., Yang M., Clarke C.H., Liao W.S., Bast R.C. (2014). ARHI (DIRAS3) induces autophagy in ovarian cancer cells by downregulating the epidermal growth factor receptor, inhibiting PI3K and Ras/MAP signaling and activating the FOXo3a-mediated induction of Rab7. Cell Death Differ..

[16] Klionsky D.J., Abdelmohsen K., Abe A., Abedin M.J., Abeliovich H., Acevedo Arozena A., Adachi H., Adams C.M., Adams P.D., Adeli K. (2016). Guidelines for the use and interpretation of assays for monitoring autophagy (3rd edition). Autophagy.

[17] Khan N.A., Nikkanen J., Yatsuga S., Jackson C., Wang L., Pradhan S., Kivelä R., Pessia A., Velagapudi V., Suomalainen A. (2017). mTORC1 Regulates Mitochondrial Integrated Stress Response and Mitochondrial Myopathy Progression. Cell Metab..

[18] Jewell J.L., Guan K.L. (2013). Nutrient signaling to mTOR and cell growth. Trends Biochem. Sci..

[19] Wang C.I., Chen Y.Y., Wang C.L., Yu J.S., Chang Y.S., Yu C.J. (2016). mTOR regulates proteasomal degradation and Dp1/E2F1- mediated transcription of KPNA2 in lung cancer cells. Oncotarget.

[20] Saxton R.A., Knockenhauer K.E., Wolfson R.L., Chantranupong L., Pacold M.E., Wang T., Schwartz T.U., Sabatini D.M. (2016). Structural basis for leucine sensing by the Sestrin2-mTORC1 pathway. Science.

[21] Wolfson R.L., Chantranupong L., Saxton R.A., Shen K., Scaria S.M., Cantor J.R., Sabatini D.M. (2016). Sestrin2 is a leucine sensor for the mTORC1 pathway. Science.

[22] Neufeld T.P. (2012). Autophagy and cell growth--the yin and yang of nutrient responses. J. Cell Sci..

[23] Sun C.C., Zhou Q., Hu W., Li S.J., Zhang F., Chen Z.L., Li G., Bi Z.Y., Bi Y.Y., Gong F.Y. (2018). Transcriptional E2F1/2/5/8 as potential targets and transcriptional E2F3/6/7 as new biomarkers for the prognosis of human lung carcinoma. Aging.

[24] Ma Y., Kurtyka C.A., Boyapalle S., Sung S.S., Lawrence H., Guida W., Cress W.D. (2008). A small-molecule E2F inhibitor blocks growth in a melanoma culture model. Cancer Res..

[25] Muller C., Calkhoven C.F., Sha X., Leutz A. (2004). The CCAAT enhancer-binding protein alpha (C/EBPalpha) requires a SWI/SNF complex for proliferation arrest. J. Biol. Chem..

[26] Wang Y., Zhu J., Zhang L., Zhang Z., He L., Mou Y., Deng Y., Cao Y., Yang P., Su Y. (2017). Role of C/EBP homologous protein and endoplasmic reticulum stress in asthma exacerbation by regulating the IL-4/signal transducer and activator of transcription 6/transcription factor EC/IL-4 receptor alpha positive feedback loop in M2 macrophages. J. Allergy Clin. Immunol..

[27] Slomiany B.A., D’Arigo K.L., Kelly M.M., Kurtz D.T. (2000). C/EBPalpha inhibits cell growth via direct repression of E2F-DP-mediated transcription. Mol. Cell Biol..

[28] Fullgrabe J., Ghislat G., Cho D.-H., Rubinsztein D.C. (2016). Transcriptional regulation of mammalian autophagy at a glance. J. Cell Sci..

[29] White E. (2015). The role for autophagy in cancer. J. Clin. Investig..

[30] Maycotte P., Thorburn A. (2011). Autophagy and cancer therapy. Cancer Biol. Ther..

[31] Levy J.M.M., Towers C.G., Thorburn A. (2017). Targeting autophagy in cancer. Nat. Rev. Cancer.

[32] Guo J.Y., White E. (2016). Autophagy, Metabolism, and Cancer. Cold Spring Harb Symp Quant. Biol..

[33] Rabinowitz J.D., White E. (2010). Autophagy and metabolism. Science.

[34] Qiu J., Li X., He Y., Sun D., Li W., Xin Y. (2018). Distinct subgroup of the Ras family member 3 (DIRAS3) expression impairs metastasis and induces autophagy of gastric cancer cells in mice. J. Cancer Res. Clin. Oncol..

[35] Wolpin B.M., Rubinson D.A., Wang X., Chan J.A., Cleary J.M., Enzinger P.C., Fuchs C.S., McCleary N.J., Meyerhardt J.A., Ng K. (2014). Phase II and pharmacodynamic study of autophagy inhibition using hydroxychloroquine in patients with metastatic pancreatic adenocarcinoma. Oncologist.

[36] Manic G., Obrist F., Kroemer G., Vitale I., Galluzzi L. (2014). Chloroquine and hydroxychloroquine for cancer therapy. Mol. Cell Oncol..

[37] Amaravadi R.K., Winkler J.D. (2012). Lys05: a new lysosomal autophagy inhibitor. Autophagy.

[38] Mao W., Peters H.L., Sutton M.N., Orozco A.F., Pang L., Yang H., Lu Z., Bast R.C. (2019). The role of vascular endothelial growth factor, interleukin 8, and insulinlike growth factor in sustaining autophagic DIRAS3-induced dormant ovarian cancer xenografts. Cancer.

[39] Wang G., Luo X., Wang J., Wan J., Xia S., Zhu H., Qian J., Wang Y. (2018). MeDReaders: a database for transcription factors that bind to methylated DNA. Nucleic Acids Res..

[40] Zhu H., Wang G., Qian J. (2016). Transcription factors as readers and effectors of DNA methylation. Nat. Rev. Genet..

[41] Estecio M.R., Yan P.S., Ibrahim A.E., Tellez C.S., Shen L., Huang T.H., Issa J.P. (2007). High-throughput methylation profiling by MCA coupled to CpG island microarray. Genome Res..

