# Supplementary figures and images for: Amino Acid Deprivation-Induced Autophagy Requires Upregulation of DIRAS3 through Reduction of E2F1 and E2F4 Transcriptional Repression
Source: Cancers (Basel). 2019 Apr 30;11(5):603. doi: 10.3390/cancers11050603 (PMC6562629; doi:10.3390/cancers11050603)

Figure 1

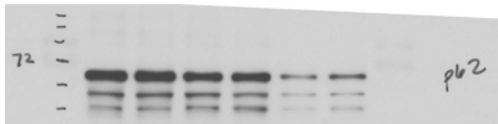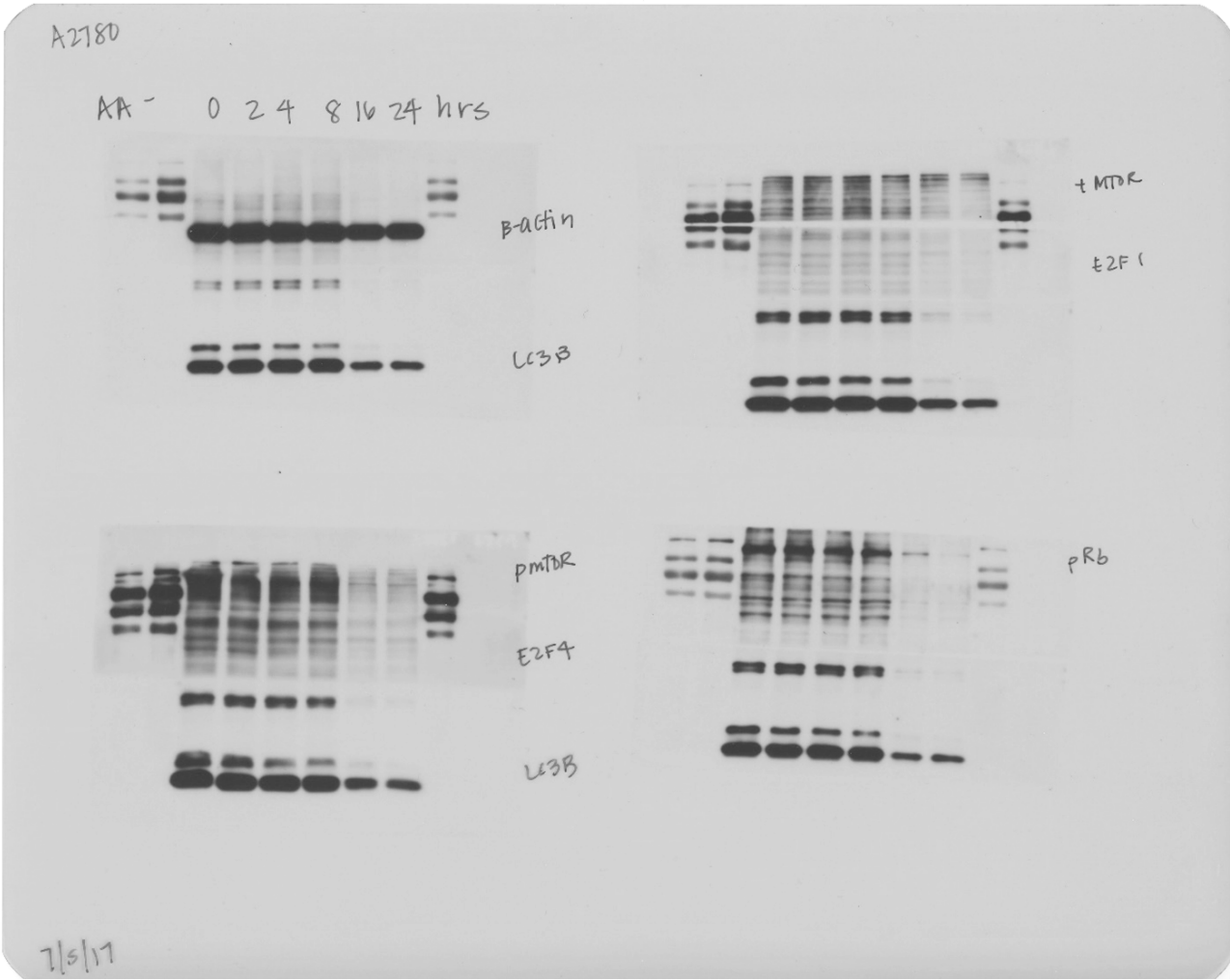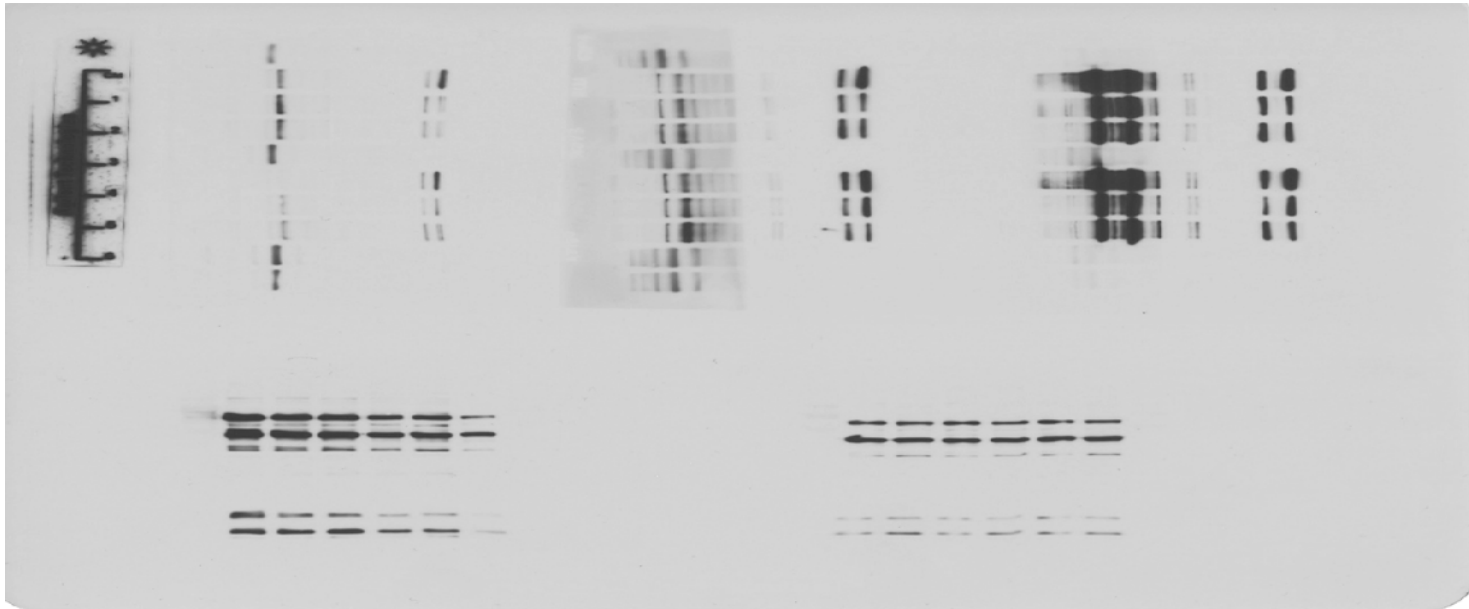

Figure 2

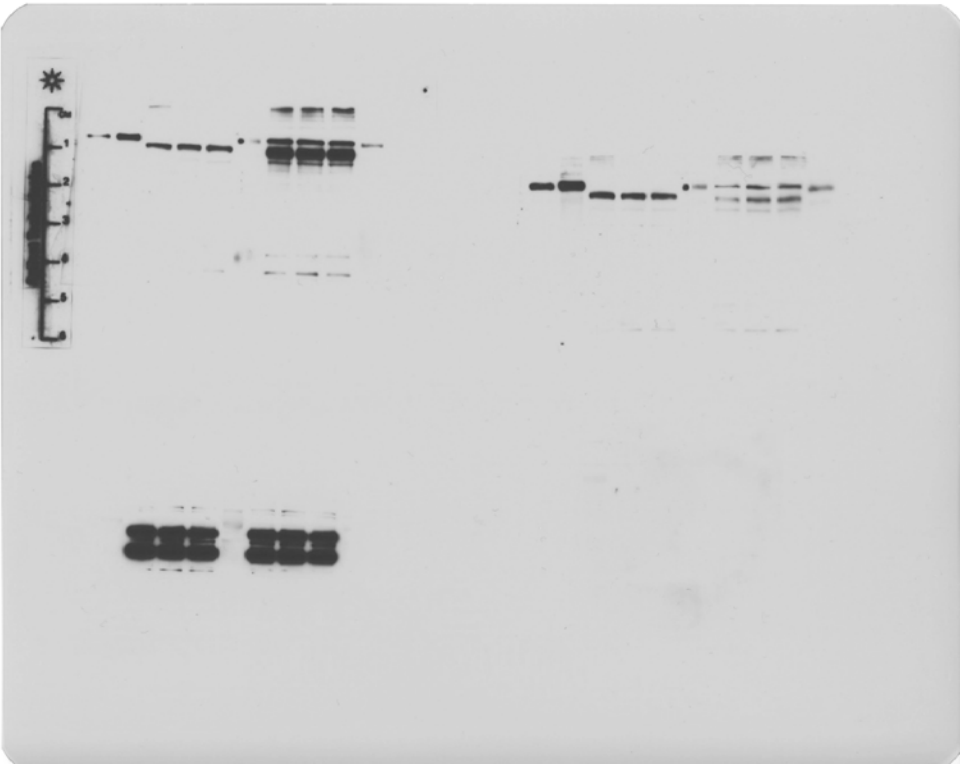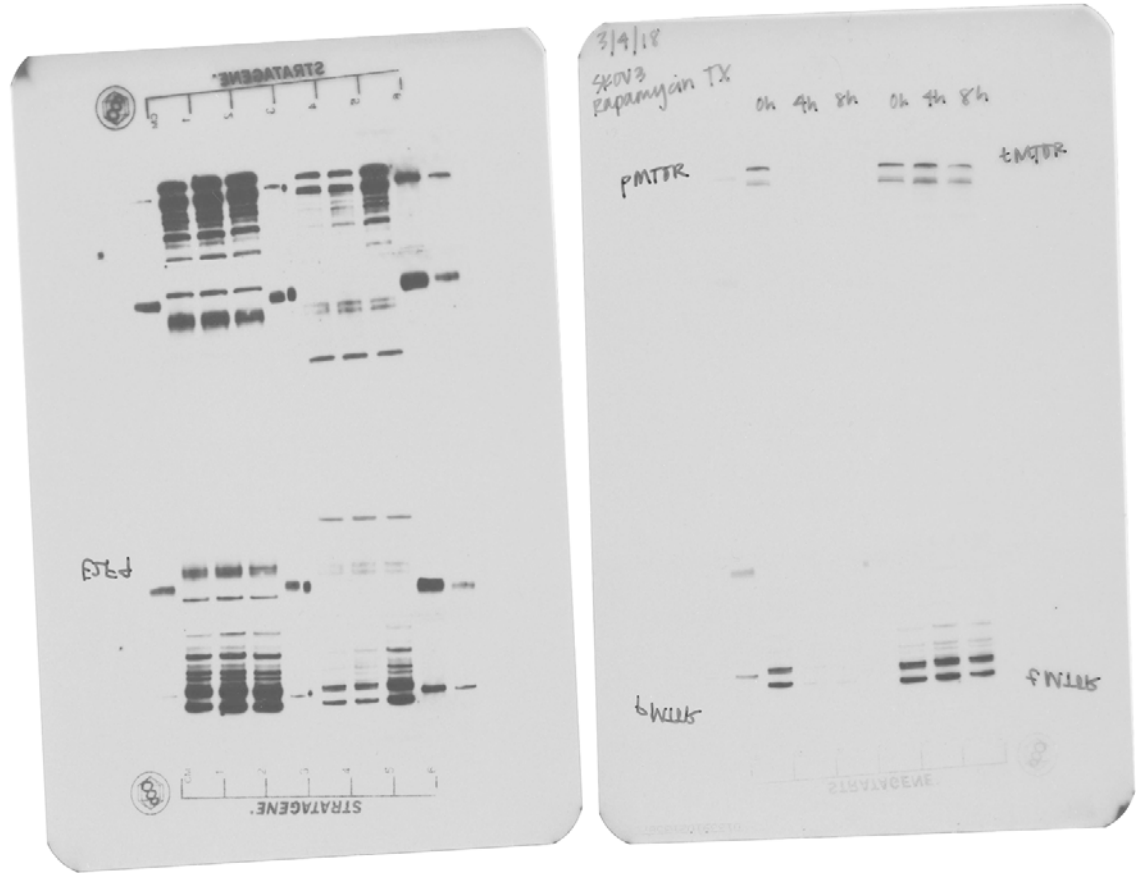

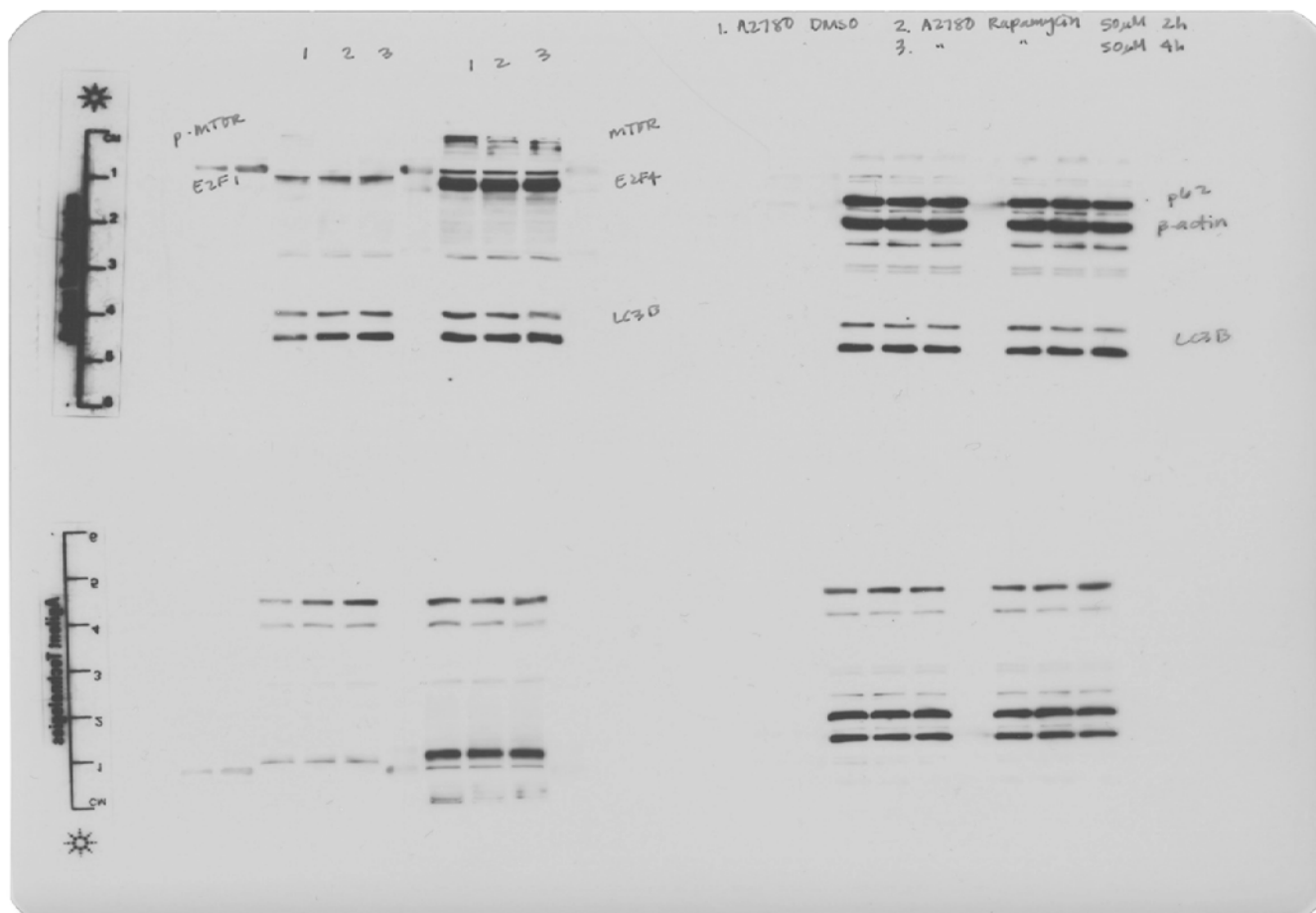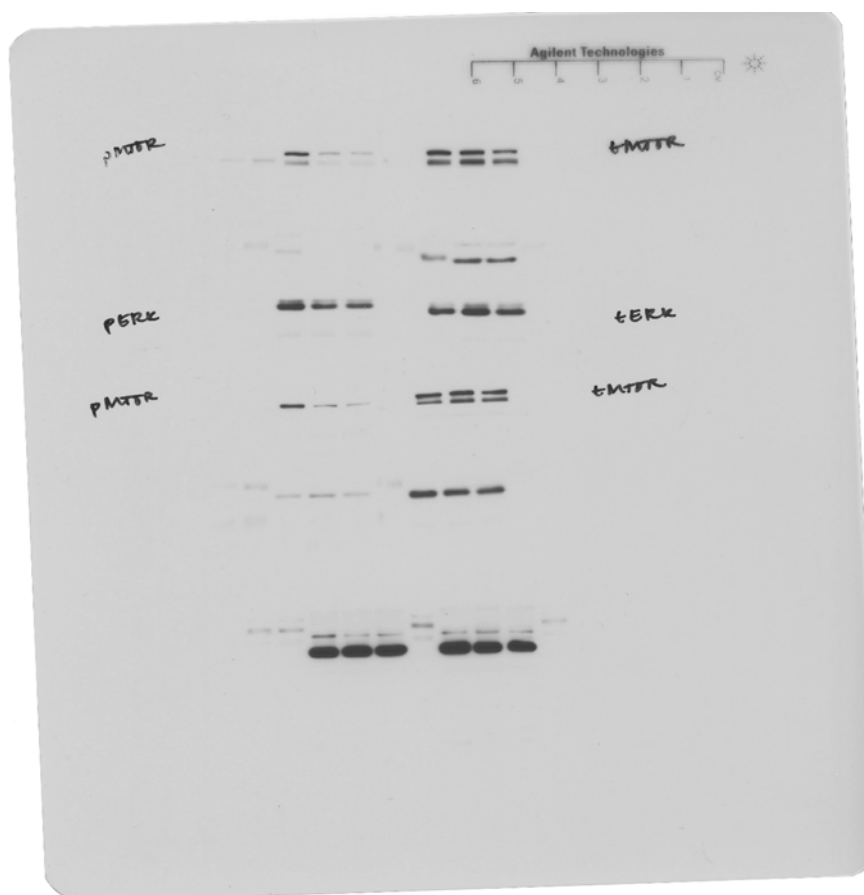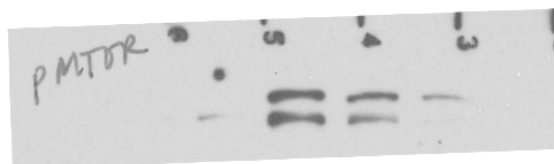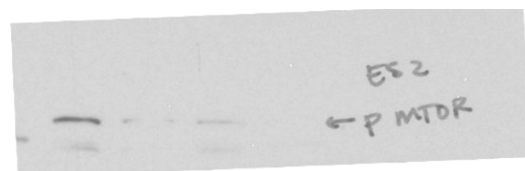

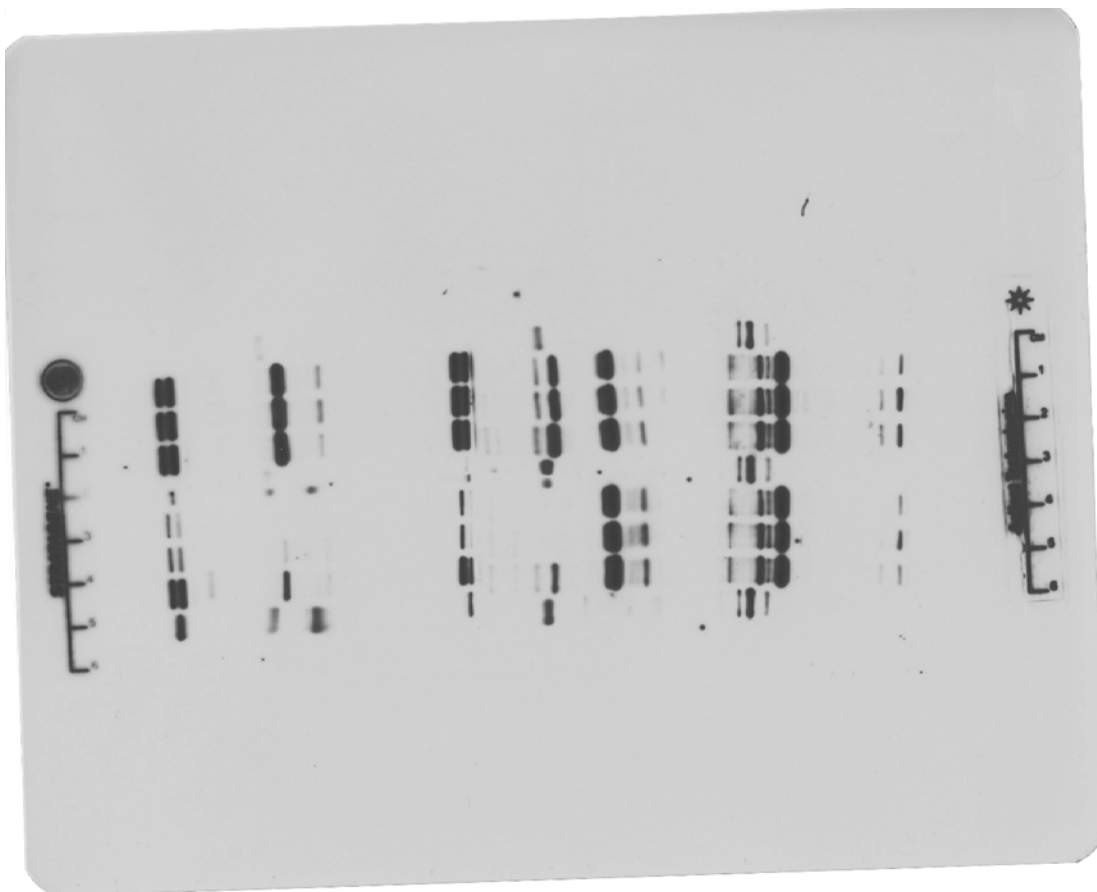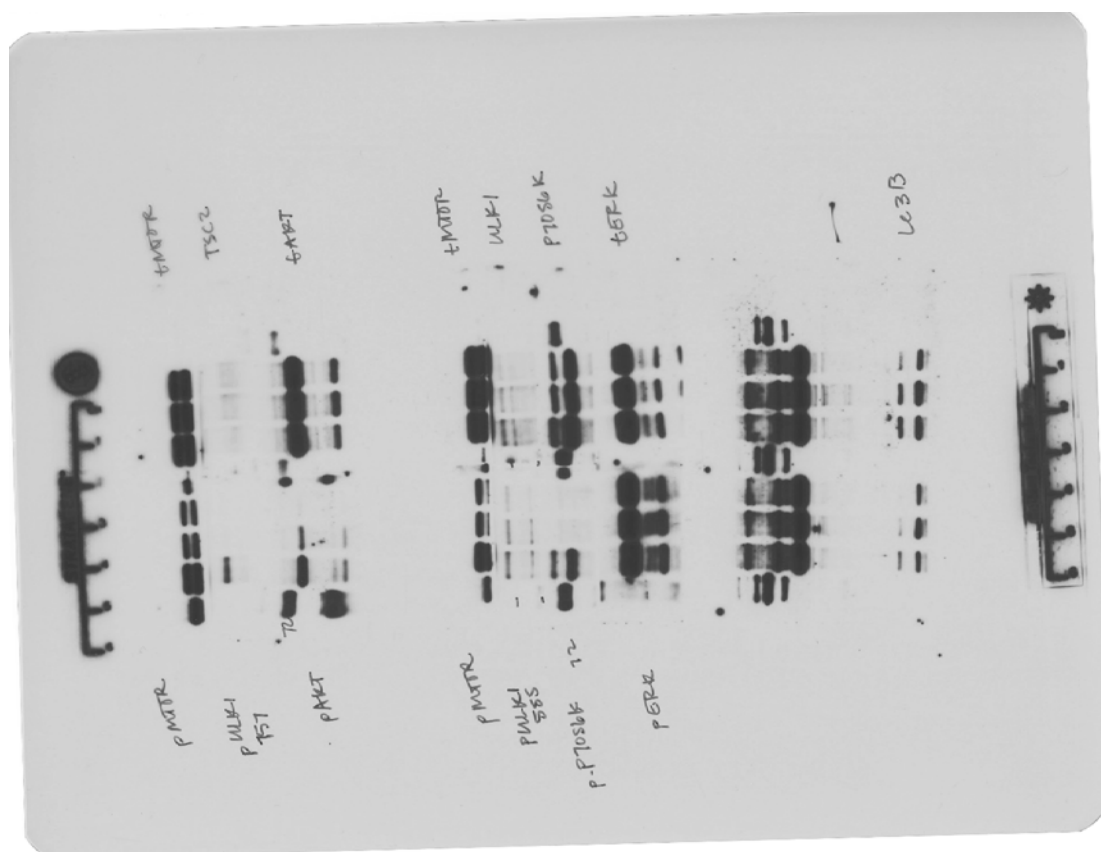

### Figure 3

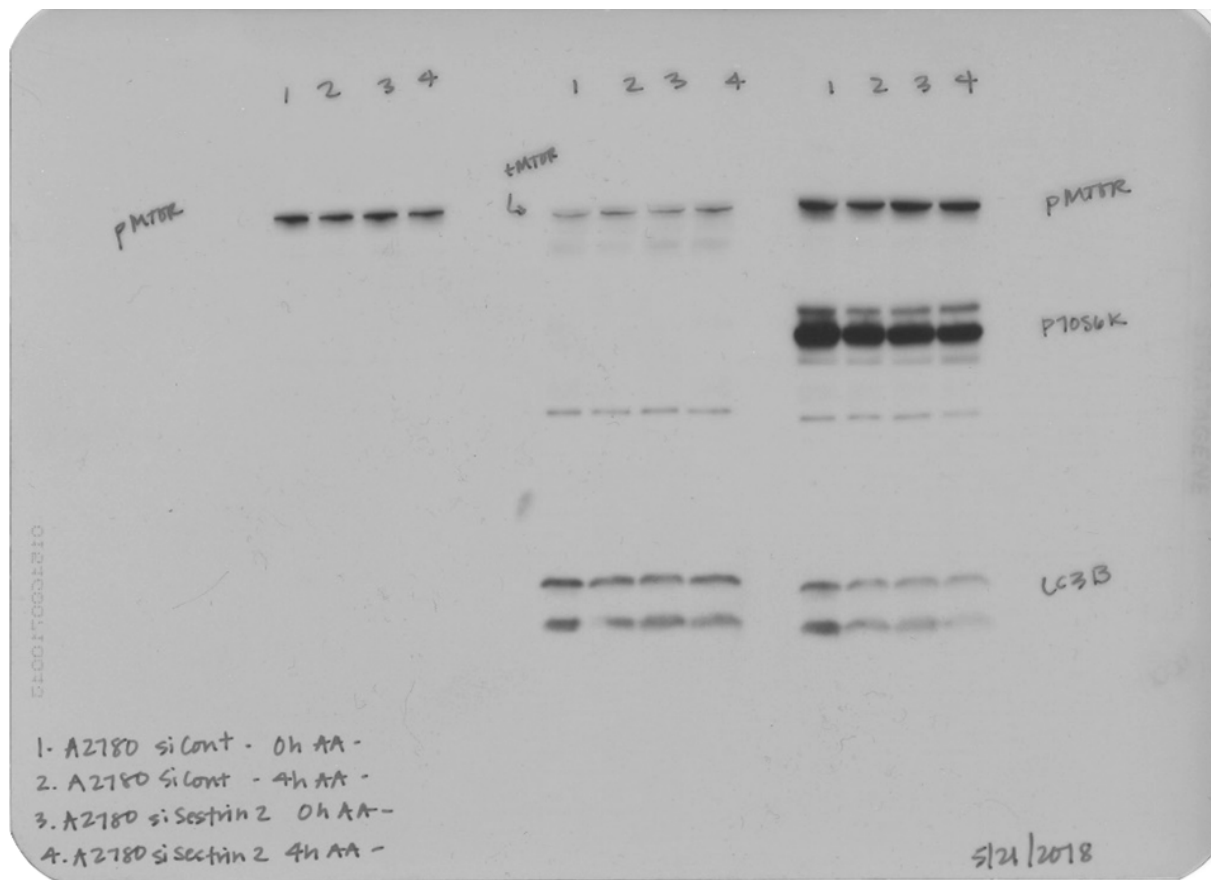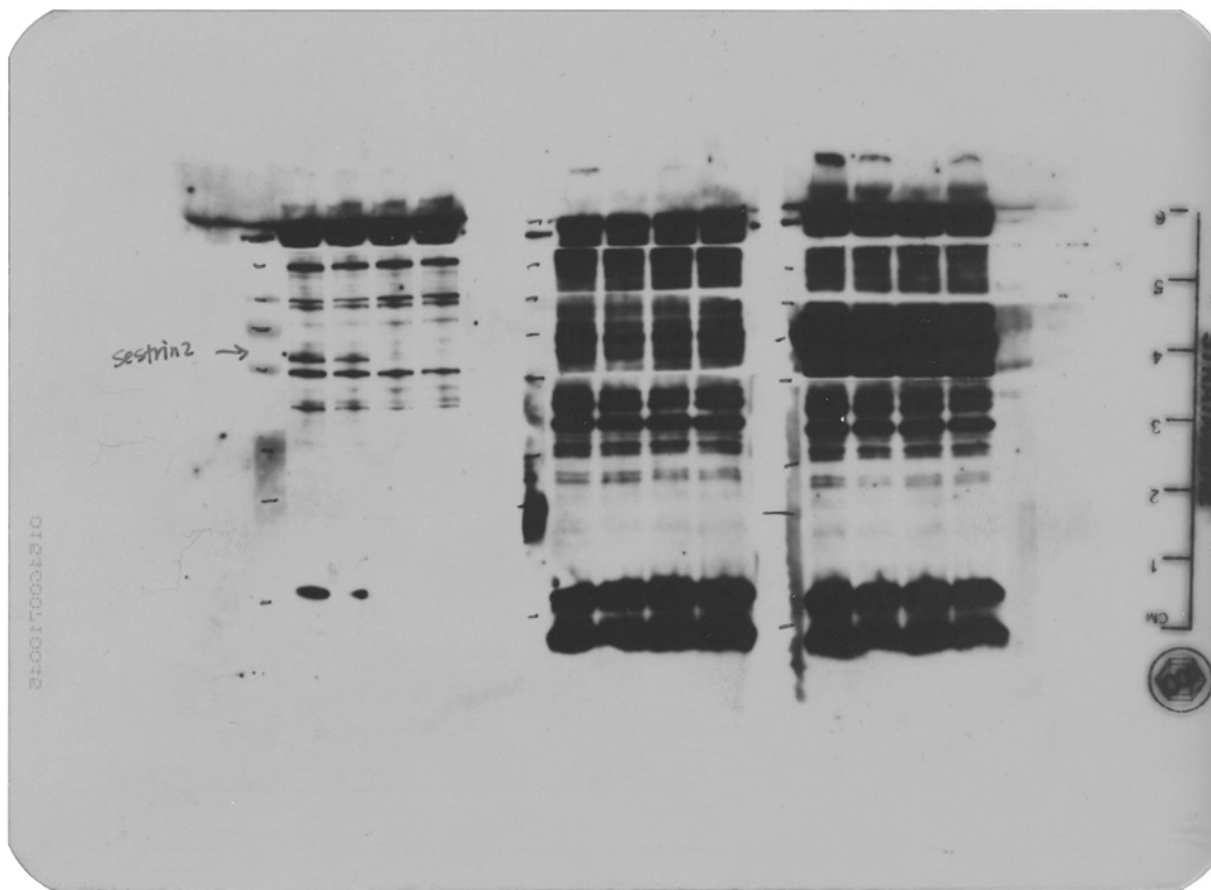

Figure 4

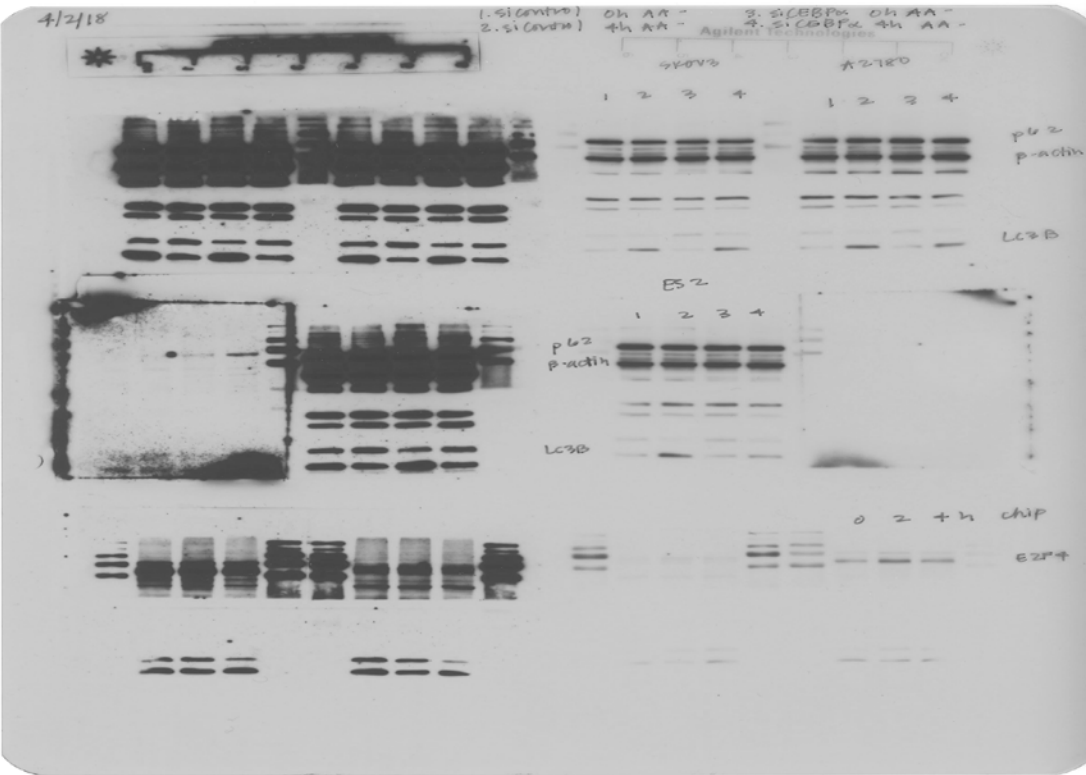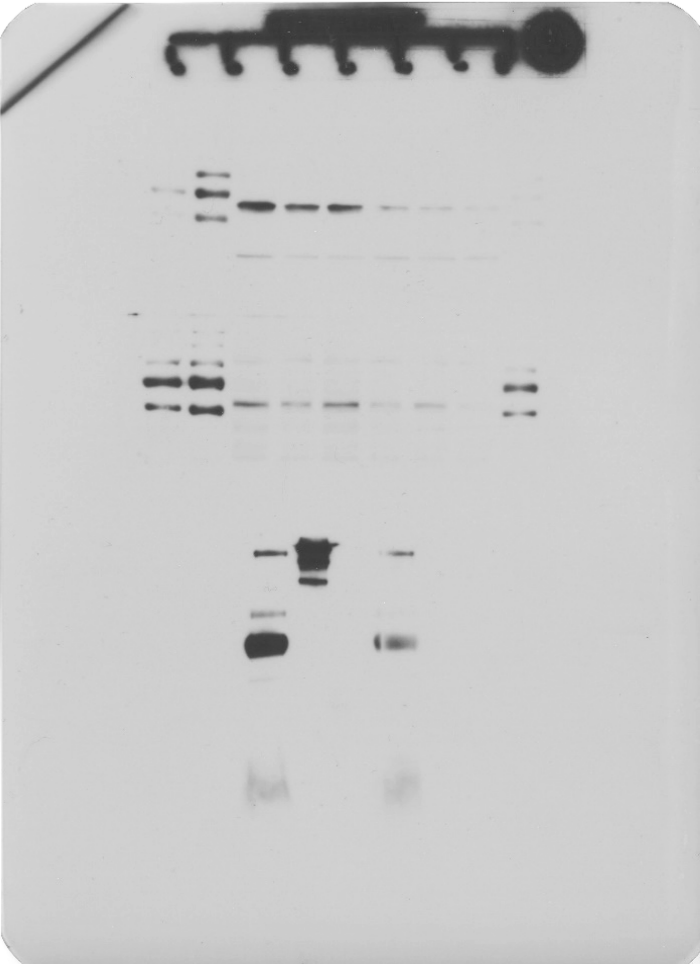

ES2

0 2 4 8 16 24

p62 →  
β-actin →

LC3B →

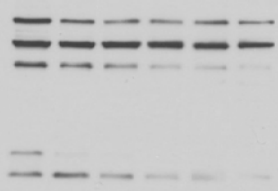

ES5

0 2 4 8 16 24

p62 →  
β-actin →

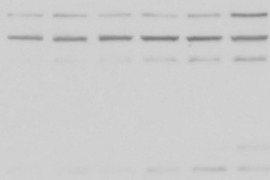

Figure 5

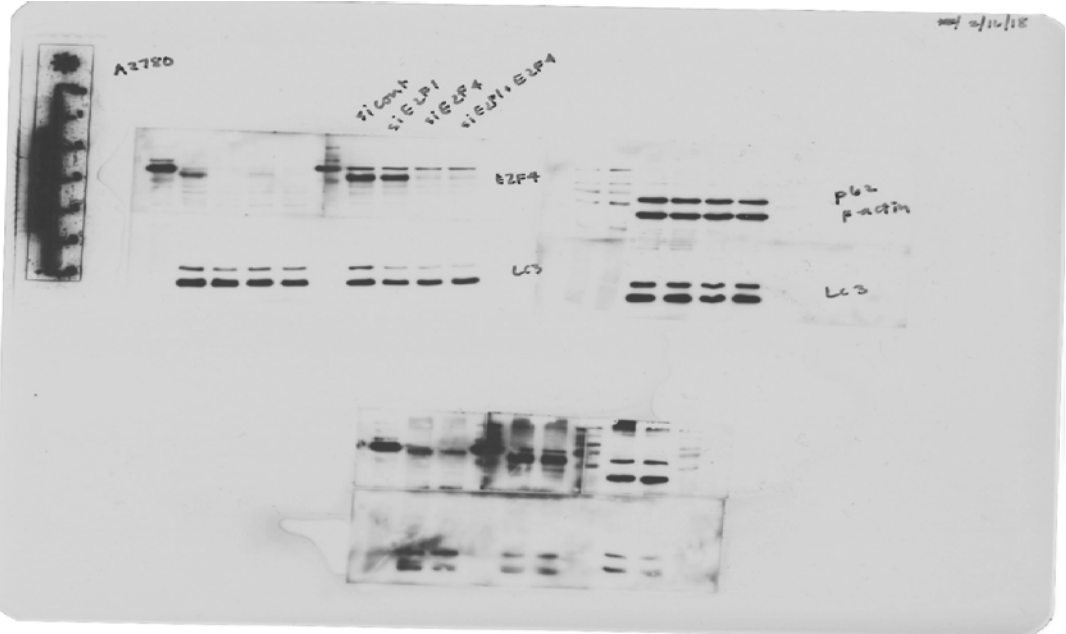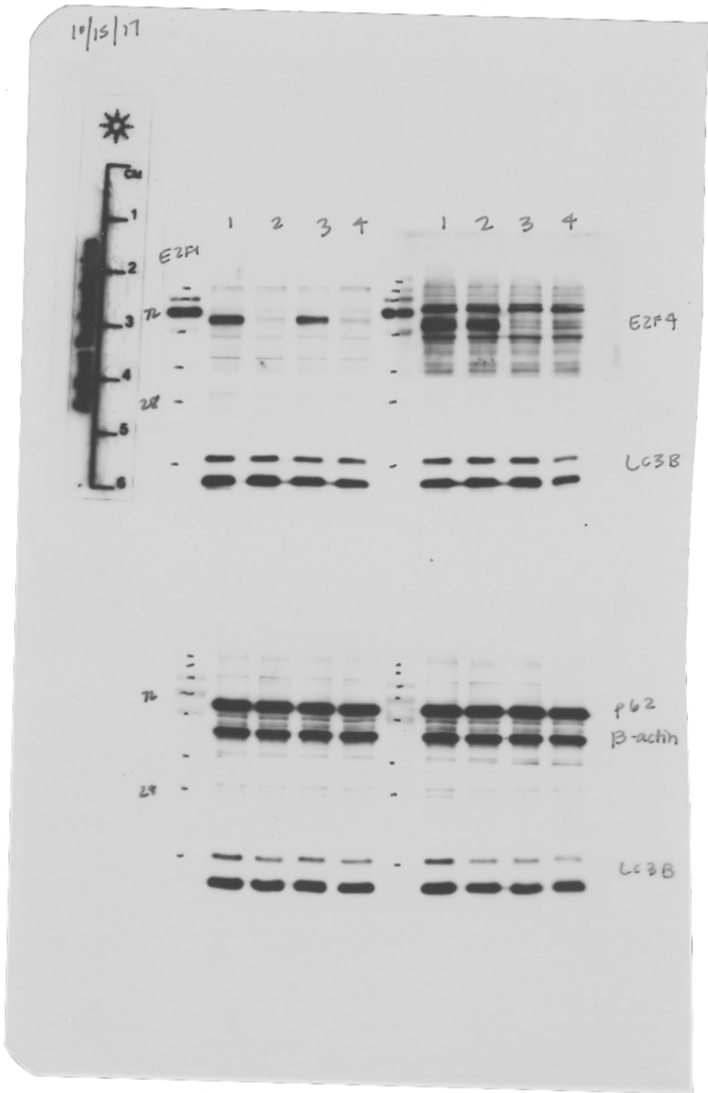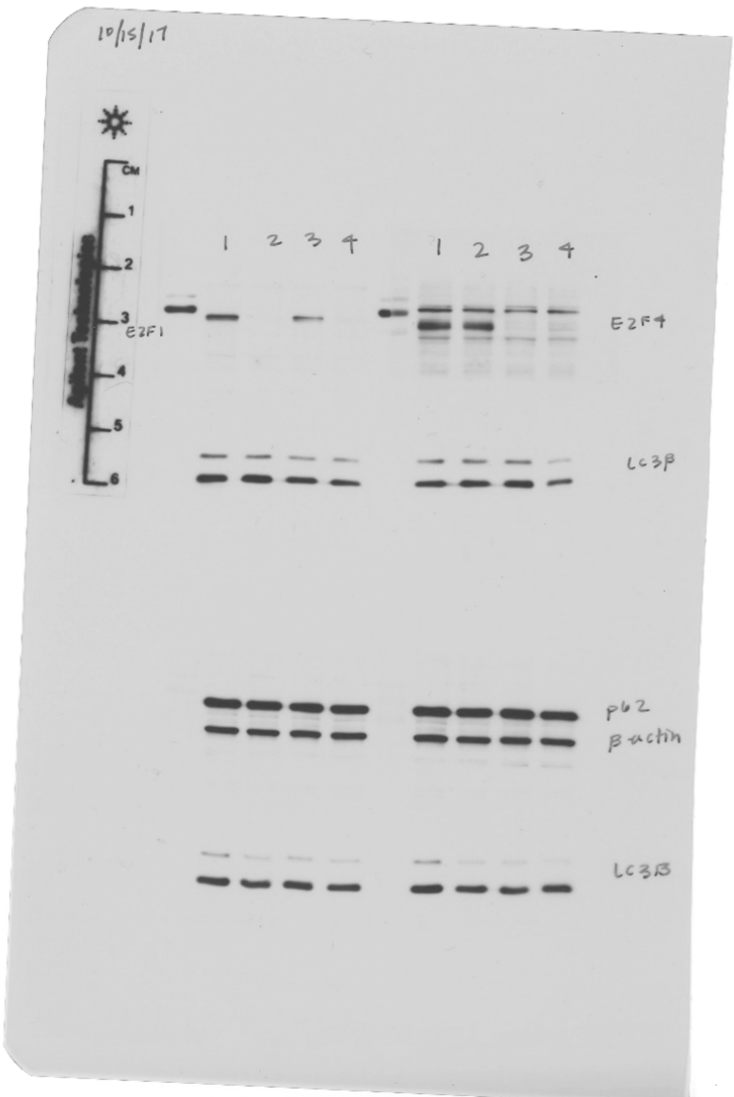

Figure 6

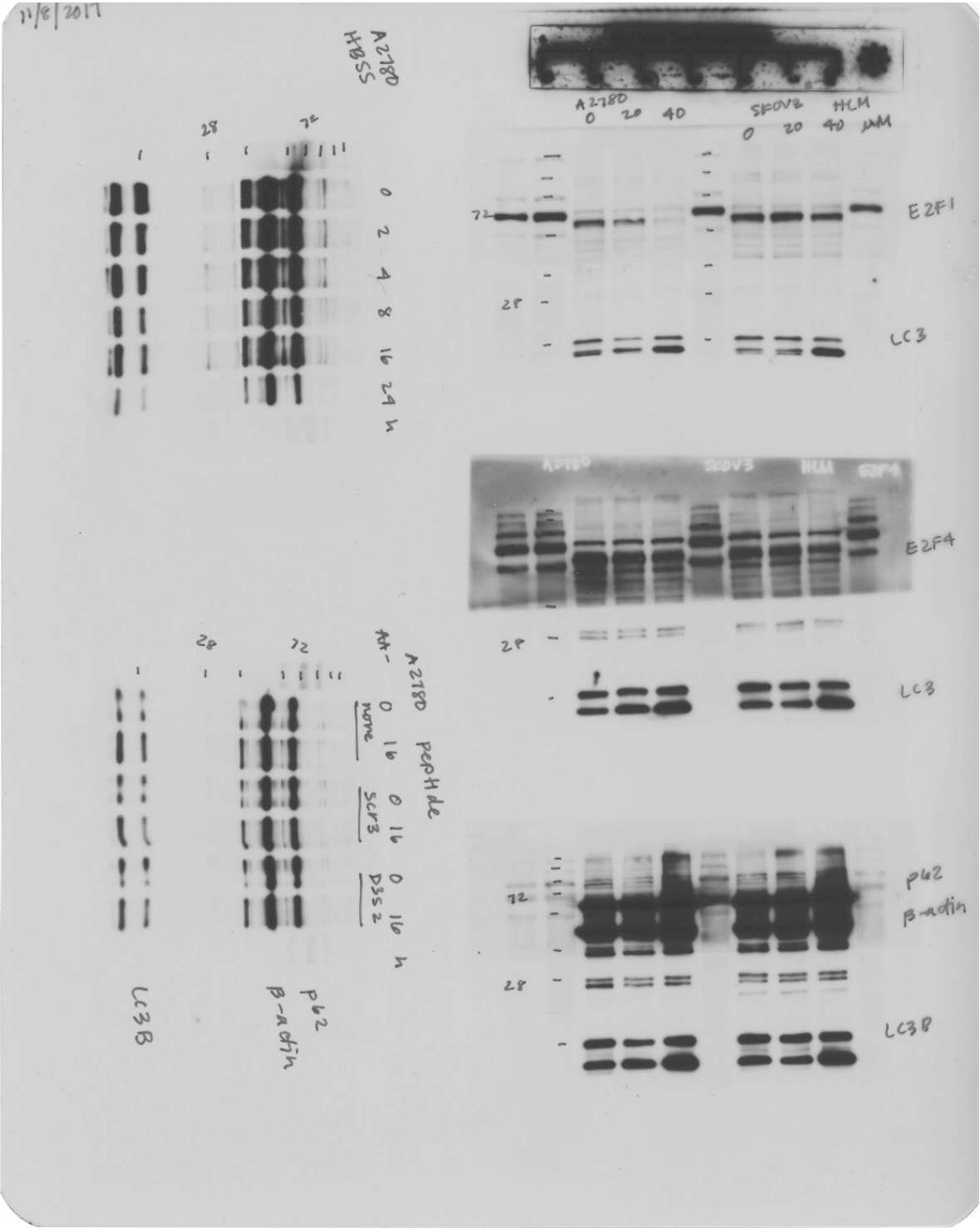

Figure 7

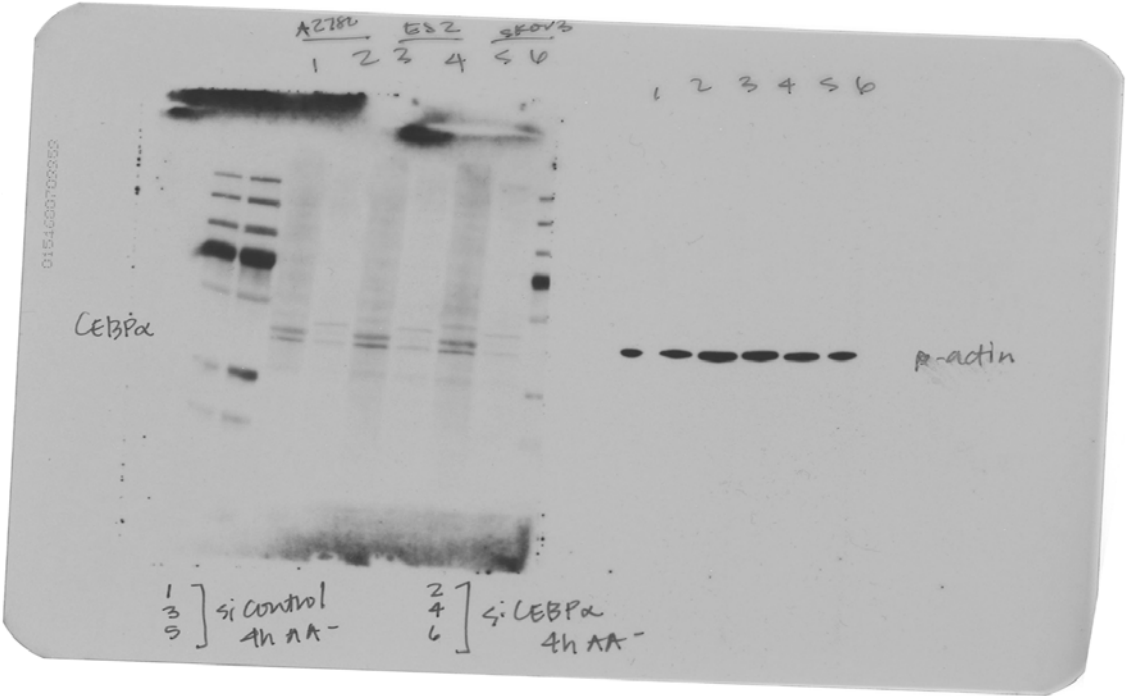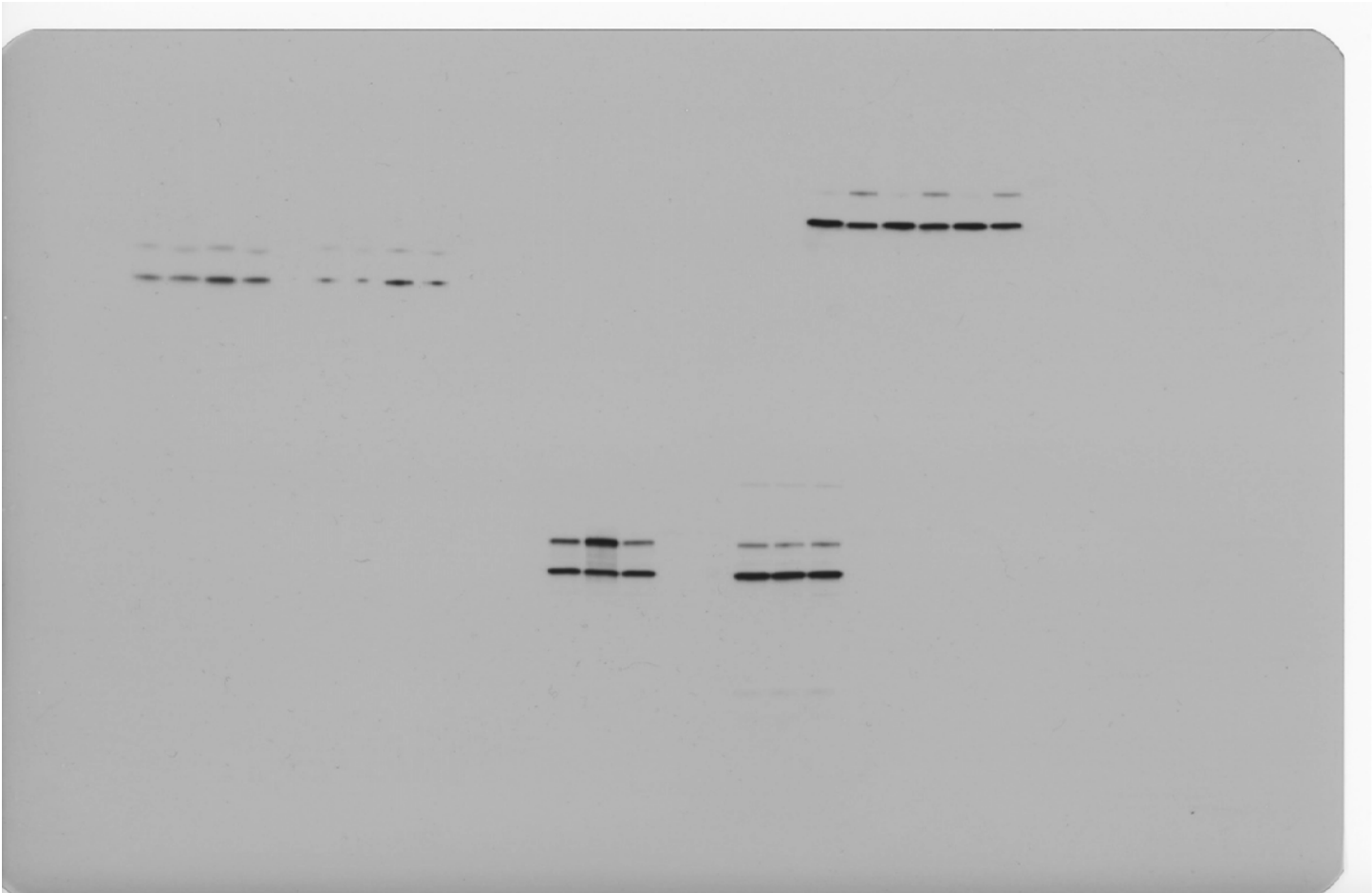

Supplement: Supplementary file 1 [file cancers-11-00603-s001.pdf]
